# Stroke secondary to cancer-associated coagulopathy: a systematic review

**DOI:** 10.1007/s00415-026-14119-y

**Published:** 2026-09-20

**Authors:** Carlota Jauregui Larrañaga, Emilio Gómez Arteta, Juan Marta Enguita, María Elena Erro Aguirre

**Affiliations:** 1 Neurology Department, Hospital Universitario Donostia, Donostia-San Sebastián, Begiristain Doktorea Pasealekua, 20014 Donostia, Gipuzkoa, Spain; 2 Biogipuzkoa Health Research Institute, Donostia-San Sebastián, Spain; 3 Hematology Department, Hospital Universitario de Navarra, Pamplona, Spain; 4 Neurology Department, Hospital Universitario Marqués de Valdecilla, Santander, Spain; 5 Neurological Clinical Research Group, Instituto Investigación Sanitaria Valdecilla (IDIVAL), Santander, Spain; 6 Neurology Department, Hospital Universitario de Navarra, Pamplona, Spain; 7 Neuroepigenetics Laboratory-Navarrabiomed, Public University of Navarre (UPNA), Navarra Institute for Health Research (IdiSNA), Pamplona, Spain; 8 Doctoral Programme in Health Sciences, Public University of Navarre (UPNA), Pamplona, Spain

**Keywords:** Stroke, Cancer, Adenocarcinoma, Hypercoagulability, Biomarkers, Anticoagulation

## Abstract

**Background:**

Stroke is the second most common neurological complication in cancer patients after metastases. Cancer-associated coagulopathy (CAC) is an important mechanism of ischemic stroke in this population. We systematically reviewed the epidemiological, clinical, radiological, and pathophysiological features of CAC-related stroke, as well as associated biomarkers and treatment strategies.

**Methods:**

A systematic search of PubMed/MEDLINE, Scopus, Cochrane Library, and Embase (2000–2025) was performed according to PRISMA 2020 guidelines. Search terms included “stroke”, “cancer”, “hypercoagulability”, “coagulopathy”, “disseminated intravascular coagulation”, and “non-bacterial or marantic thrombotic endocarditis”. Studies published in English or Spanish were included, whereas case reports and review articles were excluded. Data were synthesized qualitatively.

**Results:**

Eighty-two studies met inclusion criteria. CAC-related stroke occurs predominantly within six months of cancer diagnosis and is strongly associated with advanced or metastatic disease and a high risk of early recurrence. Adenocarcinoma, particularly lung and pancreatic cancer, is the most frequently associated histological subtype. Proposed mechanisms include mucin-mediated platelet aggregation, tissue factor-driven coagulation, extracellular vesicles, and neutrophil extracellular trap formation, leading to thromboinflammation and platelet-rich thrombi. D-dimer is the biomarker most consistently associated with recurrence and mortality, while C-reactive protein, fibrinogen, CA-125, and transcranial Doppler microembolic signals show limited specificity. Characteristic imaging findings include multiple infarcts involving more than two vascular territories, particularly the ‘three territories sign’. Low-molecular-weight heparin and direct oral anticoagulants are the most commonly used secondary prevention strategies. Thirty-day mortality rates range from 25 to 50%.

**Conclusions:**

CAC-related stroke is a distinct and underrecognized entity characterized by specific biological and radiological features and poor outcomes. Earlier recognition and optimized antithrombotic strategies may improve prognosis.

**Supplementary Information:**

The online version contains supplementary material available at https://doi.org/10.1007/s00415-026-14119-y.

## Background

Stroke is the second most common neurological complication in cancer patients after metastases [1]. *Graus *et al*.* found that 14.6% of cancer patients had cerebrovascular lesions, including hemorrhages and infarcts, at autopsy, half of which had been asymptomatic [2]. Indeed, cancer patients have twice the risk of ischemic stroke compared to controls matched for demographic variables and comorbidities [3].

Both stroke and cancer represent major public health concerns. Stroke remains the second leading cause of death worldwide and a major cause of long-term disability [4]. Meanwhile, 301,884 new cancer cases are estimated in Spain in 2026, and this incidence is expected to increase by more than 20% by 2040 [5]. As cancer incidence continues to rise and advances in oncological treatments improve patient survival, the coexistence of cancer and stroke is expected to become increasingly common. Consequently, the burden of cancer-associated stroke is likely to increase substantially in the coming decades [6].

The pathophysiological mechanisms of stroke in cancer patients are diverse and may be related to the tumor itself or to side effects of chemotherapy or radiotherapy. Cancer-associated coagulopathy (CAC) or hypercoagulability is the most frequent pathophysiological mechanism in patients with cancer and stroke, and in up to 50% of cases, no conventional etiology of stroke is identified [7, 8].

The aim of this review is to further investigate the pathophysiological mechanisms, review the clinical and radiological characteristics, and define the biomarkers of stroke secondary to CAC, in order to improve prevention, diagnosis, and treatment strategies.

## Materials and methods

### Study design

**Fig. 1:**
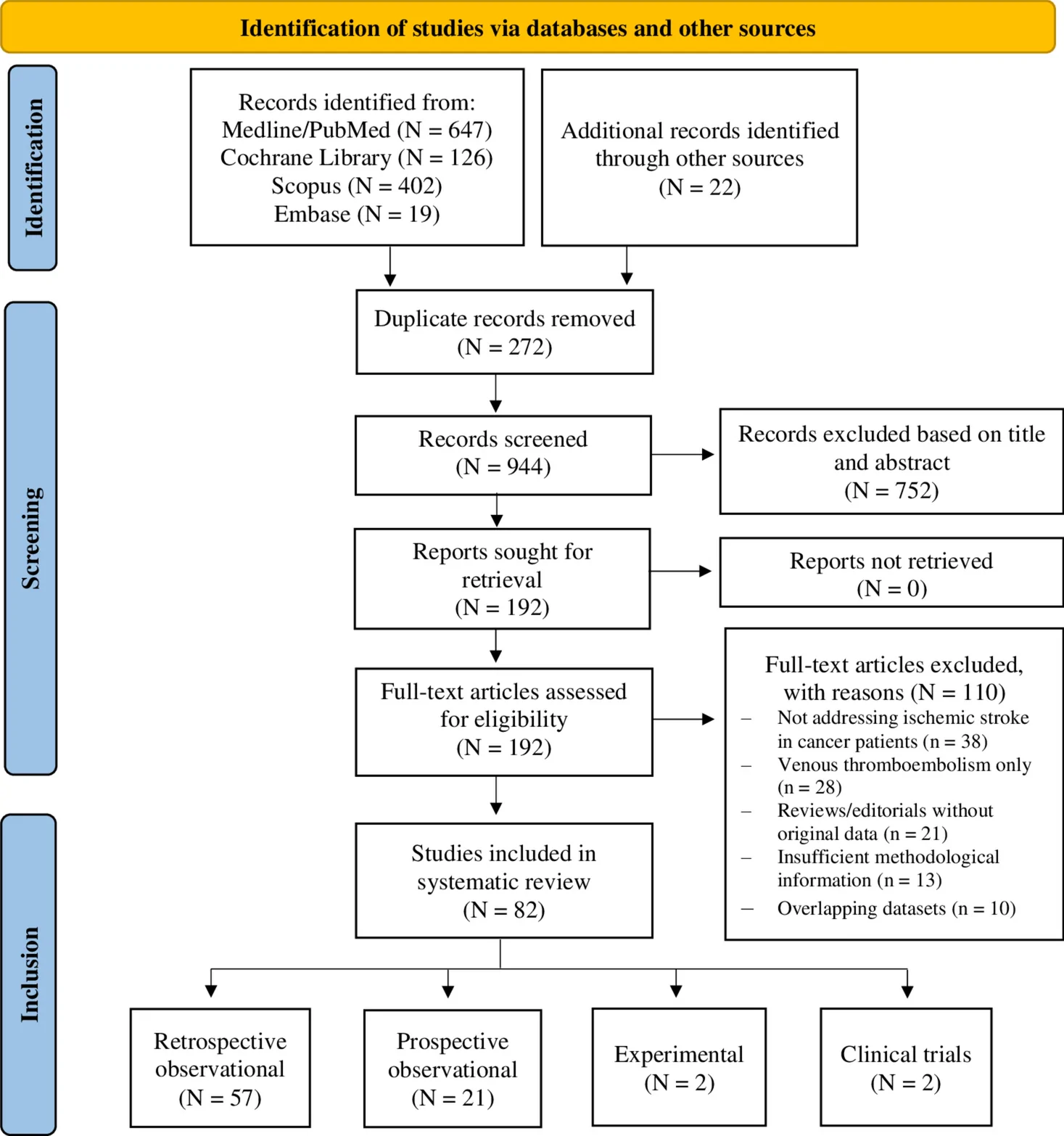
Flowchart

This study was conducted as a systematic review of the scientific literature on hypercoagulability-associated ischemic stroke in patients with cancer, following the Preferred Reporting Items for Systematic Reviews and Meta-Analyses (PRISMA) 2020 guidelines [9]. The study selection process is summarized in the PRISMA flow diagram (Fig. 1).

### Search strategy

A comprehensive literature search was performed in PubMed/MEDLINE, Scopus, Cochrane Library, and Embase databases to identify studies investigating CAC and ischemic stroke. The search strategy included combinations of the following keywords and Medical Subject Headings (MeSH): “stroke,” “cancer,” “coagulopathy”, “hypercoagulability”, “disseminated intravascular coagulation”, and “non-bacterial or marantic thrombotic endocarditis”. The full Boolean queries used for each database are provided in Supplementary Table S1 (Online Resource 1 S1).

English and Spanish filters were selected, because they represented the languages mastered by the review team. Publication year filters (from January 2000 to December 2025) were applied, but preprints were not excluded in the search strategy. Additionally, reference lists of relevant systematic reviews and original studies were screened to identify further studies for inclusion.

The database search identified 1,194 records: 647 in PubMed/MEDLINE, 402 in Scopus, 126 in Cochrane Library, and 19 in Embase. Additionally, 22 records were identified through manual screening of reference lists. In total, 1,216 records were identified through database searching and other sources.

### Study selection

Data extraction was performed independently by two reviewers using a standardized data collection form. All records were independently screened by two authors based on title and abstract. Discrepancies in study selection and data extraction were resolved by consensus.

After removing 272 duplicate records, 944 studies remained for title and abstract screening. During this phase, 752 records were excluded, because they were not relevant to the topic. A total of 192 full-text articles were subsequently assessed for eligibility.

After full-text review, 110 studies were excluded for the following reasons: studies not addressing ischemic stroke in cancer patients (*n* = 38), studies focused exclusively on venous thromboembolism (*n* = 28), review articles or editorials without original data (*n* = 21), insufficient methodological information (*n* = 13), and overlapping datasets or duplicate populations (*n* = 10).

Finally, 82 studies met the inclusion criteria and were included in the qualitative synthesis. Among these, 78 were observational studies (21 prospective and 57 retrospective), 2 were experimental studies, and 2 were randomized clinical trials.

### Eligibility criteria

Eligibility criteria were established prior to conducting the literature search. Peer-reviewed studies addressing ischemic CAC-related stroke, particularly in patients with solid tumors, were considered eligible. We have excluded studies that deal exclusively with hematological neoplasms, including conditions such as thrombotic microangiopathy, coagulopathy associated with promyelocytic leukemia, or with essential thrombocythemia. Both clinical and experimental studies investigating mechanisms, epidemiology, biomarkers, imaging features, or treatment strategies related to cancer-associated hypercoagulability were included. The following studies were excluded: case reports, review articles without original data, studies unrelated to cancer-associated ischemic stroke, and studies lacking sufficient methodological information. Duplicate records were manually removed during the screening process.

### Quality assessment

The methodological quality of the included studies was assessed according to study design. Observational studies were evaluated using the Newcastle–Ottawa Scale (NOS) [10], randomized clinical trials were assessed using the Cochrane Risk of Bias Tool Version 2 (RoB-2) [11], and experimental studies involving animal models were assessed using a version of the RoB tool developed by the Systematic Review Centre for Laboratory Animal Experimentation (SYRCLE) [12]. Individual NOS (Supplementary Table S2), RoB-2 (Supplementary Table S3), and SYRCLE (Supplementary Table S4) assessments are summarized in Online Resource 2.

Among the 82 included studies, methodological quality was overall moderate to high. According to the Newcastle–Ottawa Scale, among the 78 observational studies assessed, 46 (59.0%) scored ≥7 points, 31 (39.7%) scored 5–6 points, and 1 (1.3%) scored <5 points.

The assessment was carried out independently by two authors, and discrepancies were resolved by consensus.

The review protocol was not prospectively registered.

### Ethics approval

Ethical approval was not required for this study, because it was based exclusively on previously published data and did not involve human participants or patient-level data.

## Results

Table 1 lists the characteristics of each of the 82 articles included. The most relevant results are summarized in Supplementary Table S5 (Online Resource 3).

**Table 1:** Characteristics of included studies

Author	Year	Study design	Sample size (n)	Tumor type	Stroke subtype	Main biomarker(s)	Main imaging findings	Follow-up	Main conclusions
Taccone FS [16]	2008	Single-center retrospective cohort based on a prospectively collected stroke registry	5,106 first-ever AIS screened; 24 occult cancer (0.4%)	Multiple systemic cancers; lung and breast most frequent; metastatic 70.3%	AIS; NBTE 8/24, DIC 6/24, atherosclerosis 5/24	NR	NR	Mean 29 months (range 3–60) among survivors	First-ever stroke as the initial manifestation of cancer was rare (0.4%) and mostly related to cancer-specific mechanisms (NBTE/DIC). Most cancers were diagnosed after recurrent ischemia; mortality was 79%, with median survival of 58 days
Kim SG [25]	2010	Multicenter prospective observational cohort	161 AC + AIS: 97 CSM, 64 cryptogenic	Multiple active cancers; lung 29.2%, followed by gastric and colorectal	Cryptogenic 39.8%; CSM 60.2%	D-dimer	Multiple vascular-territory DWI lesions: 70.3% cryptogenic vs 20.6% CSM	NR	Cryptogenic/non-CSM stroke accounted for 39.8%; D-dimer >1.11 μg/mL and multiterritorial DWI lesions were independently associated with CAC
Seok JM [81]	2010	Prospective observational cohort	74 cancer + AIS; 38 without CSM, 36 with CSM	Multiple cancers; adenocarcinoma associated with embolic signals	AIS with vs without CSM	D-dimer; TCD embolic signals	NR	NR	Embolic signals were more frequent without CSM (57.9% vs 33.3%) and correlated strongly with D-dimer; higher D-dimer and adenocarcinoma independently predicted embolic signals
Shao B [51]	2011	Experimental mechanistic study (murine in vivo + ex vivo/in vitro)	Murine WT, knockout and radiation-chimera models; murine whole-blood experiments; no human clinical cohort	LS180 human colonic adenocarcinoma-derived mucins; experimental mucinous adenocarcinoma model	NA	Carcinoma mucins; P-/L-selectin; PSGL-1; cathepsin G; PAR4	NA	NA	Carcinoma mucins triggered platelet-rich microthrombosis through reciprocal neutrophil–platelet activation involving P-/L-selectin–PSGL-1 and cathepsin G–PAR4 signaling, largely independent of thrombin-mediated coagulation
Zöller B [31]	2012	Nationwide population-based retrospective cohort study	820,491 cancer patients	34 cancer types/sites**;** particularly high stroke risk with CNS tumors and leukemia; metastatic disease analyzed	Ischemic and hemorrhagic stroke	NA	NA	From cancer diagnosis until first stroke hospitalization, death, emigration or study end; <6 mo, 6–12 mo, 1–5 y, 5–10 y, ≥10 y	Cancer increased ischemic and hemorrhagic stroke risk, highest during the first 6 months after diagnosis and remaining slightly elevated ≥10 years; metastasis further increased risk
Schwarzbach CJ [26]	2012	Retrospective matched case–control analysis of prospectively collected databases	140 AIS + AC; 140 age/sex-matched controls	Multiple active solid cancers, predominantly adenocarcinoma; lung/pancreatic overrepresented; hematologic and primary brain tumors excluded	UCE 67/140 vs CSE 73/140	D-dimer	Multiterritorial infarcts 24% vs 14% controls; small embolic infarcts 34% vs 16%; multiterritorial 34% UCE vs 15% CSE	NR	Elevated D-dimer and multiterritorial/small embolic infarcts, particularly without conventional etiologies, supported CAC as an important stroke mechanism
Seok JM [45]	2012	Prospective single-center observational study	70 AC + AIS; 36 with CSM, 34 without CSM	Multiple active cancers	AIS with vs without CSM	D-dimer	PWI-DWI mismatch 58.3% CSM vs 23.5% no CSM; MRA-DWI mismatch 77.8% vs 9.7%; higher D-dimer associated with less mismatch	NR	AIS without CSM showed higher D-dimer and markedly less frequent diffusion-perfusion/MRA-DWI mismatch; stroke mechanism may influence selection for reperfusion therapy
Kono T [79]	2012	Single-center retrospective observational cohort	211 AIS: 154 without cancer, + 57 with cancer: 15 CAS, 39 CSM, 3 other	Multiple cancers; lung, stomach and liver most frequent; stage IV strongly associated with CAS	CAS vs CSM	D-dimer, FDP	NR	NR	CAS was independently associated with elevated D-dimer and FDP levels, even after adjustment for vascular risk factors and stage IV cancer
Navi BB [27]^*^	2014	Single-center retrospective cohort	263 AC + AIS	Adenocarcinoma 60%; lung 32%, GI 25%, GU 12%; systemic metastases 69%	Cryptogenic 51%; CE 22%; LAA 15%; SVO 8%; other 5%	NR	Radiographic cardioembolic/multiterritory pattern in 57% of cryptogenic strokes	Until death or July 31, 2012/last available follow-up	Cryptogenic stroke independently predicted poorer survival in AC (HR 1.64; median 55 vs 147 days), especially with a radiographic cardioembolic pattern
Navi BB [30]^*^	2014	Single-center retrospective cohort	263 AC + AIS	Adenocarcinoma 60%; lung 32%, GI 25%, GU 12%; systemic metastases 69%	Cryptogenic 51%; CE 22%; LAA 15%; SVO 8%; other 5%	NR	NR	Until death/last follow-up; 230/263 (87%) complete until death	High early recurrent thromboembolism (21%, 31%, 37% at 1, 3, 6 months) and AIS (7%, 13%, 16%); adenocarcinoma independently predicted recurrence
Guo YJ [75]	2014	Single-center retrospective cohort study	564 AIS: 98 AC, 36 inactive cancer, 430 without cancer	Multiple cancers; mainly GI 38.8% and lung 23.5%; hematological 9.2%; adenocarcinoma 55.1%; metastases 52%	AIS stratified by TOAST; undetermined etiology frequent in AC	D-dimer	Multiple vascular-territory infarctions on MRI	≥1 year after stroke or until death; 1-year mortality assessed	AC was associated with markedly elevated D-dimer and multiterritorial infarction. D-dimer ≥5.5 mg/L showed 99.6% specificity and PPV up to 93.9% for concomitant malignancy; very high D-dimer or its combination with multiterritorial infarction may prompt cancer screening
Cocho D [17]	2015	Single-center retrospective observational cohort	631 ischemic cerebrovascular events; 13 occult cancers (2.1%)	Various occult solid and hematologic malignancies	81% cerebral infarction, 19% TIA; occult cancer in undetermined etiology (5.3%)	CRP; fibrinogen	NR	NR	Occult cancer occurred in 2.1% overall and 5.3% of strokes of undetermined etiology; elevated CRP and fibrinogen may help select patients for cancer screening
Navi BB [37]	2015	Population-based retrospective matched cohort (SEER-Medicare)	327,389 cancer patients + 327,389 matched controls	Breast, colorectal, lung, pancreatic and prostate cancer	Ischemic and hemorrhagic stroke	NA	NA	From cancer diagnosis until stroke, death, or Dec 31, 2009	Incident cancer increased short-term stroke risk, particularly in lung, pancreatic, and colorectal cancer; excess risk attenuated and was generally absent beyond 1 year
Schwarzbach CJ [41]	2015	Retrospective single-center observational study of a prospectively collected cohort	32 CAS	Multiple active solid cancers; lung predominant (40%); ~90% metastatic	CAS with strong hypercoagulability and no competing conventional etiology	D-dimer >3 μg/mL; mean 15.39 ± 10.84 μg/mL	≥2 territories 84%; microembolic scattering 78%; subacute/chronic infarcts ≈50%	NR	Scattered multiterritorial DWI lesions dominated the phenotype of CAS; recurrent/subclinical infarction was frequent
Karlińska AG [49]	2015	Prospective single-center cohort	1,558 AIS; 90 cancer (41 AC, 49 NAC)	Multiple; breast, lung and colon most frequent	AC associated with undetermined etiology (62.5% vs 38.3% cancer-free)	Elevated inflammatory markers in AC	NR	NR	Active malignancy, unlike non-active malignancy, was associated with undetermined stroke etiology and greater inflammatory activity; short-term outcome was similar across groups
Jang H [91]	2015	Single-center retrospective observational cohort	79 CAS: 29 enoxaparin, 50 warfarin	Lung 48.1%; hepatobiliary 19.0%; GI 15.2%; breast/gynecologic 8.9%; other 8.9%; adenocarcinoma 68.4%	CAS	D-dimer	Multiple-territory DWI infarcts: 82.8% enoxaparin vs 78.0% warfarin	Mean 4.9 months; D-dimer reassessed at median 8 days (IQR 6–11)	Enoxaparin reduced D-dimer more than warfarin. Recurrent stroke was numerically less frequent but not significantly different; major bleeding was similar
Kneihsl M [36]	2016	Single-center retrospective cohort	300 AIS + cancer: 73 AC; 227 NAC	Multiple cancers; in AC, lung 15.1%, prostate 12.3%, liver 6.8%, pancreatic 6.8%	Cryptogenic 50.7% AC vs 32.5% NAC	CRP	Multiple vascular-territory infarcts: 26.0% AC vs 5.2% NAC	NR	AC was associated with more severe and more frequently cryptogenic stroke, multi-territory infarction and higher in-hospital mortality (21.9% vs 6.2%); AC independently predicted in-hospital death (OR 3.70)
Gon Y [7]	2016	Retrospective single-center observational study of a prospectively collected stroke database	1,191 AIS; 145 AC (68 cryptogenic and 77 known mechanisms)	Multiple active cancers; adenocarcinoma 63%; systemic metastases 42%	Cryptogenic 47%**,** LAA 16%, AF 14%, SVO 5%, NBTE 4%, other mechanisms	D-dimer	Multiple vascular lesions: 71% cancer-associated vs 16% non-cancer cryptogenic stroke	NR	In cryptogenic stroke, higher D-dimer (OR 6.30) and multiple vascular lesions (OR 6.40) independently predicted AC, supporting a distinct hypercoagulable, multiterritorial phenotype
Thålin C [62]	2016	Prospective observational matched case–control study	41: 31 AIS (12 high hsTnT + 19 normal hsTnT controls) + 10 healthy controls	8 AC; 7/8 adenocarcinomas of different origins	AIS stratified by high vs normal hsTnT	H3Cit, hsTnT, G-CSF, TAT, soluble P-selectin	Multiple/disseminated cerebral lesions beyond a single vascular territory: 50% cancer vs 4% non-cancer	2 months	Cancer was markedly enriched among AIS patients with high hsTnT; NETosis was linked to widespread systemic arterial microthrombosis involving brain and myocardium, supporting NET-related markers as indicators of cancer-associated arterial thrombosis
Bang OY [57]	2016	Prospective observational cohort (OASIS-CANCER)	313: 155 AC + AIS (116 CAS, 39 CSM), 25 AIS without cancer, 32 cancer without stroke, 101 healthy controls	Among CAS: lung 48.3%, hepatobiliary 20.7%, GI 12.9%, breast/gynecologic 10.3%, others 7.8%	CAS vs CSM	Cancer cell-derived EVs (CD326/EpCAM +); TF+ EVs; D-dimer	NR	NR	Cancer cell-derived EVs were increased in CAS and correlated with D-dimer; path analysis supported a TF-independent pathway linking cancer-cell EVs, coagulopathy and ischemic stroke
Finelli PF [71]	2016	Single-center retrospective imaging-based observational study	41 patients with ≥3-territory DWI lesions identified from 4,075 MRI studies; 9 malignancy-related infarctions	Among 9 CAS: lung 4, colon 2, renal 1, pancreatic 1, bladder 1	CAS in 9/41 (22%)	NR	Three-territory DWI pattern involving bilateral anterior + posterior circulations; typically peripheral, non-enhancing/non-ring-forming, 0.5–2 cm or larger-territory lesions, usually non-watershed	NR	Malignancy accounted for 22% of three-territory DWI infarctions and 75% of cases without an identifiable embolic source; this pattern should prompt consideration of CAC
Shin YW [89]	2016	Single-center retrospective cohort	93 AC + cryptogenic AIS	Multiple cancers; lung 22%, GI 18%, pancreatic 15%; adenocarcinoma 87%; metastases 80%	Cryptogenic AIS/CAS	D-dimer	Multiple vascular-territory DWI lesions in 85%	Mean observation 151 days; surviving patients followed ≥225 days	Overall survival was very poor (median 62 days [IQR 32–223]); high D-dimer, systemic metastases, and diabetes independently predicted mortality, with high D-dimer approximately doubling mortality risk (HR 2.01)
Lee MJ [34]	2017	Prospective observational cohort (OASIS-CANCER)	268 AIS + AC	Lung 39.2%; GI 19.4%; hepatobiliary/pancreatic 18.7%; others 22.8%; adenocarcinoma 66.4%; metastases 65.7%	Cryptogenic 71.6%; conventional 28.4%	D-dimer	NR	Overall and 1-year survival; median follow-up 2,302 days among survivors	Higher pretreatment D-dimer independently predicted poorer overall and 1-year survival; persistent post-treatment hypercoagulability predicted mortality, whereas successful correction of D-dimer after anticoagulation was associated with improved 1-year survival
Nam KW [84]	2017	Two-center retrospective observational cohort	109 cryptogenic AIS + AC; 34 END (31%)	Multiple solid cancers	Cryptogenic AIS	D-dimer	Multiple vascular-territory lesions	3 months; END assessed within 72 h; follow-up MRI in 72 patients	Higher D-dimer independently predicted END (aOR 1.11) and was associated dose-dependently with new-territory infarction, supporting recurrent thromboembolism as a mechanism of early deterioration
Cutting S [99]	2017	Single-center retrospective cohort	49 cancer + AIS	Multiple cancers; lung 36.7%, prostate 14.3%, breast 10.2%; metastatic disease 53.1%; brain tumors/metastases excluded	CAC 42.9%; CE 18.4%; cryptogenic 16.3%; other mechanisms	NR	NR	3 months	Three-month mortality was 46.9%; 50% of 3-month survivors had poor functional outcome. Admission NIHSS was the principal independent predictor of poor functional outcome
Selvik HA [18]	2018	Prospective registry-based observational cohort (NORSTROKE)	1,646 AIS; 82 AC (5.0%)	Prostate 22.0%, lung 20.7%, colon 15.9%, bladder 12.2%, gynecological 4.9%, pancreatic/gastric/melanoma 3.7% each	AIS; undetermined etiology independently associated with AC	D-dimer; hemoglobin	NR	12 months after index stroke for cancer ascertainment	AC occurred in 5% of AIS. D-dimer, low Hb, smoking, and undetermined stroke etiology independently associated with AC; a score using D-dimer ≥3 mg/L, Hb ≤12 g/dL, and smoking yielded a 53% post-test probability when all 3 points were present
Quintas S [8]	2018	Single-center longitudinal retrospective cohort	381 AIS; 29 occult cancers (7.6%)	Colon 24.1%, lung 13.8%, prostate 13.8%, hematologic 13.8%; adenocarcinoma 62.1%; metastatic 34.5%	AIS; no significant etiologic differences between cancer/non-cancer groups	Fibrinogen; hemoglobin	Multiple vascular territories: 17.2% cancer vs 7.4% non-cancer (p=0.062)	18 months	Occult cancer was diagnosed in 7.6%, at a median of 6 months after AIS. Older age, CKD, previous cancer, lower Hb, and higher fibrinogen were associated with subsequent cancer diagnosis, but only smoking remained independently associated in multivariable analysis
Grazioli S [15]	2018	Multicenter retrospective cohort	2,209 AIS; 98 AC (4.4%)	Prostate 15.6%, hematologic 15.6%, lung 13.7%, colorectal 11.7%, breast 7.8%, others	AIS; Cryptogenic stroke independently associated with AC (OR 1.93)	LDL-C	NR	NR; in-hospital outcomes only	Age >65 years, VTE, LDL-C <70 mg/dL, and cryptogenic stroke independently associated with AC. In-hospital mortality was higher with AC (21.5% vs 10%), supporting an important role for hypercoagulability
Chung JW [58]	2018	Prospective observational cohort (OASIS-CANCER substudy)	114 active lung cancer + AIS; 95 adenocarcinoma; 95 distant metastasis	Lung cancer. adenocarcinoma 83.3% vs non-adenocarcinoma 16.7%; distant metastases 83.3%	AIS undetermined	Cancer-cell EVs (EpCAM/CD326 +); D-dimer	Larger acute infarct volume in adenocarcinoma	NR	Lung adenocarcinoma was associated with larger infarct volumes, higher circulating cancer-cell EV levels, and greater coagulopathy; EVs from adenocarcinoma showed greater procoagulant activity than those from squamous carcinoma
Wang J [74]	2018	Retrospective case–control study	137 AIS: 61 cancer, 76 non-cancer controls; D-dimer available in 53 cancer patients	Multiple cancers; gastric, lung and colorectal most frequent; AC 70.5%, metastatic 47.5%	AIS with cancer vs AIS without cancer	D-dimer, FDP	AMBIs involving ≥3 vascular territories and both anterior/posterior circulations	NR	CAS showed higher D-dimer/FDP and more multiterritorial infarction. D-dimer >2.785 μg/mL identified a marked hypercoagulable phenotype; ≥3-territory and anterior–posterior circulation infarcts were characteristic imaging findings
Nam KW [87]	2018	Two-center retrospective observational cohort	85 cryptogenic AIS with AC; 15 END	Multiple solid cancers; hepatobiliary most frequent, followed by lung, gastric, GI and GU; hematologic malignancies excluded	Cryptogenic AIS with AC	NLR	Larger initial DWI infarct volume associated with END	3 months; serial NLR through day 7; END ≤72 h	Higher NLR at days 1–3 independently predicted END (aOR 2.78); patients with END subsequently had worse 3-month functional outcomes, supporting a relationship between early inflammatory activation and neurological progression
Navi BB [94]	2018	Pilot open-label randomized clinical trial (TEACH)	20 AC + AIS: 10 enoxaparin, 10 aspirin	19 solid tumors, 1 hematologic; adenocarcinoma 9/20; systemic metastases 18/20	Undetermined 75%; LAA 20%; CE 5%	NR	NR	12 months; assigned treatment for 6 months, visits at 1, 3 and 6 months	The trial demonstrated feasibility of randomization but was limited by very small sample size and 60% crossover from enoxaparin to aspirin. No meaningful efficacy conclusions or significant differences in major bleeding, thromboembolism or survival could be established
Xiang E [90]	2018	Single-center retrospective cohort	214 cancer patients receiving anticoagulation: 77 enoxaparin, 66 warfarin, 71 DOAC	87.4% solid tumors: lung 28.3%, breast 14.5%, GU 8.6%, colorectal 6.4%, pancreatic 5.3%; 12.7% hematologic	NA	NR	NA	NR	DOAC use was associated with fewer bleeding events and/or discontinuations than enoxaparin, but major/minor bleeding and thromboembolic events did not significantly differ between DOACs, enoxaparin, and warfarin. Evidence is indirect for CAS
Park H [65]	2019	Multicenter prospective cohort-based propensity-matched comparative histopathological study	48 MT thrombi: 16 AC, 16 NAC, 16 no cancer controls	Multiple cancers: colorectal 25%, hematologic 18.8%, gastric/esophageal 12.5%, GU 12.5%, lung 12.5%, others	AIS treated with MT	Platelets (CD42b), erythrocytes (glycophorin A), fibrin/fibrinogen; NETs/neutrophils	NA	NR	Thrombi from active-cancer stroke were platelet-rich and erythrocyte-poor; this phenotype was especially marked in NBTE and cryptogenic active-cancer stroke, supporting a major role of platelet-mediated thrombogenesis
Bang OY [61]	2019	Prospective observational cohort (OASIS-CANCER)	138: 38 CAS, 27 cancer controls, 40 AIS controls, 33 healthy controls	Multiple active cancers	CAS	Plasma DNA, nucleosomes (NET biomarkers), D-dimer	NR	NR	Plasma DNA and nucleosome levels were elevated in CAS and correlated with D-dimer; higher circulating DNA was independently associated with CAS and hypercoagulability, supporting NETosis as a mechanistic pathway
Nouh AM [73]	2019	Single-center retrospective comparative cohort study	231 AIS: 64 known malignancy, 167 AF without malignancy	Multiple cancers; lung 40%, GI 33.3%, others 26.7%	Malignancy-associated vs AF-related AIS	NR	TTS: bilateral anterior and posterior circulation DWI infarcts	NR	TTS occurred in 23.4% of CAS vs 3.5% of AF-related AIS; sensitivity 23.4% and specificity 96.4%. TTS should prompt evaluation for malignancy when no alternative etiology is identified
Ren R [28]	2020	Single-center retrospective comparative cohort study	416 AIS + malignant tumor; 212 stroke-first, 204 tumor-first	Multiple malignant tumors; metastatic status analyzed	CAS/TS; stroke-first vs tumor-first presentation	Coagulation parameters	NR	NR	Cerebral infarction was the initial manifestation in 51%. Metastatic cancer (OR 2.52) and depressive state (OR 3.16) independently predicted stroke-first presentation; coagulation status did not differ between groups
Zhang J [21]	2020	Single-center case–control study	129 AIS: 51 active lung cancer + AIS, 78 AIS without cancer	Active lung cancer	AIS; SUE and SOE predominated in lung-cancer group	D-dimer; FDP	Multiterritorial infarcts, frequently involving both anterior and posterior circulations	90 days	Lung cancer-associated AIS showed greater hypercoagulability (higher D-dimer/FDP), more acute multiterritorial brain infarction, and worse 90-day prognosis; 80.4% occurred within 1 year of lung-cancer diagnosis
Yoo J [47]	2020	Retrospective analysis of a prospective hospital-based stroke cohort	245 AIS + AC: 20 NBTE, 96 cryptogenic, 129 determined etiology	Multiple active solid cancers; all NBTE patients had metastatic cancer (100%); hepatobiliary 30%, pancreatic 20%, gastric/esophageal 15%	NBTE 8.2%; cryptogenic 39.2%; determined etiology 52.7%	D-dimer	Multiple vascular-territory infarction in 90% of NBTE	6 months	NBTE was confined to metastatic cancer and carried very poor prognosis: 6-month mortality 80% and recurrent stroke 50%; NBTE independently predicted the composite of death/recurrent stroke (HR 1.94)
Fu CH [64]	2020	Retrospective observational cohort	152 EVT thrombi: 19 AC, 107 CE, 26 LAA	Multiple cancers	AIS undergoing EVT	Fibrin/platelets; RBCs; WBCs	NA	NR	AC thrombi had markedly higher fibrin/platelet content; fibrin/platelet composition was independently associated with CAS, and ≥65% discriminated CAS with AUC 0.84
Guo L [69]	2020	Retrospective observational cohort	108 cryptogenic stroke patients; 12 OSM, 96 non-OSM	OSM	Cryptogenic stroke	D-dimer	Number of vascular territories involved on DWI-MRI (> 1 territory)	6 months after discharge for occult cancer ascertainment	Higher D-dimer and greater number of involved DWI territories independently predicted OSM. A territory-based score showed good discrimination (AUROC 0.84); involvement of >1 territory should prompt extensive cancer screening
Bao L [70]	2020	Single-center retrospective matched comparative cohort	211: 31 TS-related AIS + 180 controls (80 LAA, 60 CE, 40 SAO)	All adenocarcinomas; gastric 29%, lung 22.6%, ovarian/colon 9.7% each, other solid cancers	TS-related AIS vs CSM	D-dimer; CEA; CA125; CA19-9	Multiple DWI lesions in multiple vascular territories in 87.1%	30 days	TS-related stroke was characterized by markedly elevated D-dimer and tumor markers, predominantly adenocarcinoma, and multiterritorial DWI infarcts; it was associated with greater stroke severity, disability, and mortality than conventional stroke subtypes
Kawano T [86]	2021	Retrospective hospital-based cancer-registry cohort	18,217 cancer patients; 69 with AIS	Multiple cancers; brain tumors excluded	AIS	NLR	NA	2 years after cancer diagnosis	Higher NLR at cancer diagnosis independently predicted incident ischemic stroke within 2 years; NLR >15 was associated with markedly higher stroke risk than NLR <5
Maezono-Kandori K [85]	2021	Single-center retrospective comparative cohort	**7**7 AIS + AC; 45 hypercoagulation, 32 non-hypercoagulation	Multiple; lung 25%, pancreatic 17%; pancreatic cancer more frequent in hypercoagulation group (27% vs 3%); metastases 73%	CAS vs non-hypercoagulation AIS	CA125; D-dimer	Multiple vascular-territory infarcts	NR	Elevated CA125 was independently associated with CAC (aOR 5.59), supporting CA125 as a potential biomarker
Nakajima S [35]	2022	Multicenter retrospective observational cohort	282 CAS	Multiple; lung 27.0%, pancreatic 12.1%, hepatobiliary 10.3%, colorectal 8.5%; adenocarcinoma 61.7%; metastases 50.7%	CAS	D-dimer	Multiple DWI infarcts 68.4%	30 days	Post-treatment D-dimer ≥10 μg/mL, but not pretreatment D-dimer, independently predicted poor functional outcome and mortality at 30 days; recurrent AIS was 9.9% and mortality 12.4%
Nezu T [29]	2022	Prospective multicenter observational cohort	120 AIS + AC; 47 CAS	Various active cancers	CAS 47/120 vs non-CAS 73/120	CA-125, D-dimer, CRP	Multiple DWI lesions in multiple vascular territories	NR	CA125 was the tumor marker most strongly associated with CAS (highest quartile OR 2.91), correlated with D-dimer and CRP, and together with multiterritorial DWI lesions may help identify CAS
Navi BB [48]	2022	Prospective two-center matched cohort (MOST-Cancer)	150: 50 AC + AIS, 50 matched AIS-only, 50 matched cancer-only; prognostic analysis in 50 AC + AIS	Multiple solid tumors; lung 28%, pancreatic 22%, breast 16%; adenocarcinoma 88%; distant metastases 86%	AC+AIS: undetermined 48%, CE 36%, LAA 10%, other determined 6%, SVD 0%	D-dimer, P-selectin, sICAM-1, sVCAM-1; TCD microemboli**;** TAT and thrombomodulin	NR	Median 48 days (IQR 18–312)	Thromboembolism/death occurred in 86% (recurrent thromboembolism 56%, recurrent AIS 26%). D-dimer, P-selectin, sICAM-1, sVCAM-1, and TCD microemboli predicted thromboembolism/death; D-dimer was the only marker associated with recurrent AIS
Kataoka Y [63]	2022	Single-center retrospective histopathological comparative cohort based on a prospective stroke registry	180 EVT-treated AIS: 17 cancer, 163 non-cancer	Adenocarcinoma 14/17 (82%); colorectal 3, gastric 2, pancreatic 2, prostate 2, others	AIS treated with EVT; cancer vs non-cancer	Fibrin/platelet fraction, erythrocyte fraction, D-dimer	NA	3 months	Cancer thrombi were fibrin/platelet-rich and erythrocyte-poor. Fibrin/platelet >55.7% combined with D-dimer >1.3 mg/dL was strongly associated with cancer (OR 7.06), suggesting that clot histology may help identify occult malignancy
Finelli PF [72]	2022	Single-center prospective observational cohort	22 TTS: 16 CAC, 4 infectious endocarditis, 2 indeterminate	Cholangiocarcinoma 3; lung 3; pancreatic 3; NSCLC 2; breast 1; carcinoid 1; ovarian 1; lung mass not biopsied 1; unknown primary 1;	Three-territory DWI stroke; CAC in 16/22 (72.7%)	D-dimer	TTS: acute DWI lesions involving bilateral anterior and posterior circulations	NR	CAC was the predominant etiology of TTS (16/22); malignancy was occult in 9/16 CAC cases. TTS was frequently overlooked clinically/radiologically and often heralded occult malignancy
Tanimura J [40]	2022	Single-center retrospective cohort	29 analyzed; 26 complete follow-up	GI cancers 73% (pancreas/colon/bile duct predominant); adenocarcinoma 53.8%; stage IV 96.2%	CAS: ≥2 vascular territories, active cancer, no alternative etiology	Serum albumin; D-dimer	Three vascular territories involved in 80.8%	NR	CAS was associated with advanced cancer and very short survival (median 44.5 days [IQR 27.3–76.8]); higher serum albumin predicted longer survival, whereas stroke severity and D-dimer did not
Costamagna G [24]	2023	Single-center retrospective registry-based cohort (ASTRAL)	6,686 AIS; 362 AC: 102 newly diagnosed cancer, 260 known active cancer	Various; GI 27%, lung 23%, GU 21% most frequent	CAS 152/362 (42.5%)	NR	NR	12 months	AC was present in 5.4% of AIS and newly diagnosed cancer in 1.5%. Hypercoagulability was the main cancer-related mechanism; newly diagnosed cancer had higher 1-year mortality than known AC (HR 2.11), whereas recurrent stroke risk was similar
Bravo-Anguiano Y [38]	2023	Single-center retrospective observational study with case–control analysis	616 AIS; 46 AC (7.5%)	Multiple; digestive 19/46, GU 12/46, breast 5/46; other hematologic/solid cancers	AIS with vs without AC	Hb; hematocrit; fibrinogen; CRP	>1 vascular territory: 15.8% AC vs 4.4% non-AC	3 months	AC was associated with multiterritorial ischemia, lower Hb/hematocrit, and higher fibrinogen/CRP. Functional outcome and mortality tended to be worse at 3 months, but no adequate independent predictive model for AC was obtained
Cen G [23]	2023	Multicenter prospective matched comparative study	342: 38 HCC-related cerebral infarction, 152 HCC without AIS, 152 AIS without HCC	HCC	HCC-related cerebral infarction; all HCRCI were cryptogenic by TOAST	TEG-MA; D-dimer, AFP, neutrophil count	Scattered lesions 84.2%; multiple vascular regions 68.4%; bilateral anterior + posterior circulation 39.5%	NR	HCC-related cerebral infarction showed marked hypercoagulability; higher TEG-MA, D-dimer, AFP, and neutrophil count were independently predicted AIS. MA ≥61.35 had the best discrimination (AUC 0.875)
Feldman S [55]	2023	Retrospective genomic cohort study	11,871 solid cancers; 160 ATE events, including 106 AIS	Multiple solid tumors; 74% metastatic	ATE (106/160 AIS)	KRAS, STK11 somatic mutations	NA	Up to 1 year	KRAS (HR 1.98) and STK11 (HR 2.51) alterations independently increased ATE risk, irrespective of cancer type; similar associations were seen for ischemic stroke
Beyeler M [77]	2023	Single-center retrospective cohort based on prospective stroke registry	2,256 AIS with thrombus imaging; 161 AC (7.1%), including 36 occult	Multiple active cancers	AIS with symptomatic intracranial occlusion	D-dimer, CRP, leukocytes, hemoglobin, fibrinogen	Absence of SVS on MRI; absence of HVS on CT	NR	Absence of SVS, but not HVS, was independently associated with AC (aOR 3.14). SVS may provide an imaging clue to cancer-related hypercoagulability but had low sensitivity
Gon Y [104]	2023	Prospective multicenter observational cohort (SCAN)	135 AC + AIS; 65 cryptogenic, 70 known etiologies	Lung 16%; colorectal 14%; pancreatic 13%; prostate 9%; gastric 8%; metastasis 51%	Cryptogenic 48%; known etiologies 52%	D-dimer; hs-CRP	Multivascular-territory infarcts: 64% cryptogenic vs 29% known etiologies	1 year	Distant metastasis, D-dimer, and DVT/PE independently predicted mortality. Cryptogenic stroke predicted worse survival only in univariable analysis
Ohno M [100]	2023	Single-center retrospective observational cohort	48 cancer + AIS; 24 cryptogenic, 24 known mechanisms	Lung 9, pancreatic 9, hepatobiliary 7, colorectal 5; others	Cryptogenic 50%; CE 16.7%; SVO 16.7%; atherosclerosis 12.5%; other 4.2%	D-dimer	NR	3 months after stroke or last follow-up	Cancer + AIS was associated with severe functional impairment and short survival, especially in cryptogenic stroke; median survival was 34 vs 590 days for cryptogenic vs known mechanisms
Fujinami J [83]	2023	Two-center retrospective cohort	360 AIS + AC; 73 with CAS	Pancreatic 30%; lung 19%; gastric 12%; ovarian 10%; biliary 8%; adenocarcinoma 71%	CAS	D-dimer post/pre-treatment ratio	Three-territory DWI sign 78%	Median 28 days (IQR 11–65)	Persistent D-dimer elevation after anticoagulation independently predicted recurrent stroke (HR 2.20 per 0.1 increase in post/pre ratio); ratio ≥0.80 had 92% sensitivity and 78% specificity
Gon Y [20]	2024	Retrospective multicenter population/registry-based cohort	97,448 cancer patients; 2,159 developed ATE	91,369 solid; 6,079 hematologic malignancies	ATE: AIS, myocardial infarction, peripheral arterial occlusion; TIA excluded	NA	NA	Up to 5 years after cancer diagnosis; median 3.85 years (IQR 1.43–5.00)	ATE risk was highest early after cancer diagnosis and associated with male sex, older age, advanced stage, and hematologic malignancy. AIS accounted for 71.6% of ATE; ATE was associated with approximately doubled mortality
Acir I [39]	2024	Single-center retrospective observational study	201 cancer-related stroke assessed; 153 included	Multiple; breast 17%, colon 15%, lung 14.4%, bladder 7.2%, gastric/prostate 6.5%, others	CAS	D-dimer, CRP, ESR; coagulation parameters (aPTT, PT, INR)	NR	NR	Hematologic/coagulation profiles differed according to cancer type; D-dimer, CRP, and ESR varied significantly, and stroke occurred earlier after colon and pancreatic cancer, supporting cancer-specific differences in prothrombotic biology
Patrzalek P [32]	2024	Single-center retrospective cohort	115 AC + echocardiographically confirmed NBTE	Lung 39.1%, pancreatic 16.5%, gynecologic 14.8%, GI 8.7%, hematologic 8.7%	NBTE-related systemic arterial embolism; AIS in 94/115 (81.7%) at presentation	NR	NA	Longitudinal after NBTE diagnosis; 2-year event estimates	Cancer-associated NBTE predominantly involved lung and digestive cancers and frequently presented with cerebral embolism. Despite anticoagulation, 2-year recurrent ATE was 15.9%, recurrent stroke 12.4%, and mortality 77.9%
Cen G [50]	2024	Single-center prospective matched cross-sectional comparative study	48: 16 CAS, 16 AC without stroke, 16 AIS without cancer	Adenocarcinoma 50%: lung 50%; nasopharyngeal 18.8%; liver 12.5%; colorectal, bladder and esophageal 6.3% each	CAS	NLR, PLR, ICAM-1, D-dimer	Multiple cerebral infarcts: 87.5% CAS vs 25% stroke-only	NA	CAS showed increased NLR, PLR, ICAM-1, and D-dimer and frequent multiple MRI infarcts, supporting inflammation, endothelial injury, and hypercoagulability as contributing mechanisms
Fukunaga D [78]	2024	Single-center retrospective observational study	691 initially assessed; 208 LVO analyzed (10 CAS, 198 CE)	Multiple; lung and pancreatic most frequent	CAS vs CE	D-dimer	Absent SVS: 90% CAC vs 24% CE; TTS: 90% vs 3%	NR	Absence of SVS was independently associated with CAC (OR 43), with 90% sensitivity, 78% specificity, and 99% NPV for distinguishing CAH from CE
Chung JW [95]	2024	Multicenter prospective randomized open-label blinded-endpoint pilot trial (ENCHASE)	40 cancer-related ESUS; 20 edoxaban, 20 enoxaparin	Multiple active cancers; adenocarcinoma 77.5%; systemic metastases 57.5%	Cancer-related ESUS	D-dimer; TCD microembolic signals	NR	90 days	Edoxaban and enoxaparin produced comparable reductions in D-dimer and microembolic signals; the pilot was underpowered for clinical efficacy/safety outcomes
Navi BB [96]	2024	Post hoc subgroup analysis of a multicenter double-blind RCT (ARCADIA)	137 with history of cancer: 61 apixaban, 76 aspirin	Multiple cancer types	Cryptogenic AIS with atrial cardiopathy	NA	NA	Median 1.5 years (IQR 0.6–2.5)	Among patients with a history of cancer, major ischemic/hemorrhagic events occurred in 13.1% with apixaban vs 21.1% with aspirin, but the difference was not significant (HR 0.61, 95% CI 0.26–1.43); clinically important benefit remains uncertain
Kawano T [102]	2024	Prospective multicenter observational cohort (SCAN)	90 AC + AIS; 46 high-vWF, 44 low-vWF	Lung 18%; pancreatic 13%; colorectal 13%; breast/prostate 8% each; other	Cryptogenic ~53%; other mechanisms	vWF antigen; D-dimer; hs-CRP	Multiple infarct lesions: 62% high-vWF vs 27% low-vWF	1 year	High vWF was associated with greater stroke severity, cryptogenic etiology, VTE, elevated D-dimer/hs-CRP, and multiple lesions, and independently predicted 1-year mortality (OR 6.77)
Zhao B [33]	2024	Single-center retrospective descriptive case series	21 TS + AIS	Adenocarcinoma 17/21 (80.95%): lung 4, gastric 4, prostate 3, pancreatic 2, colorectal 2; breast 2; other	TS–related AIS	D-dimer	Multivascular infarction in 19/21 (90.5%); multiregional patchy DWI lesions	90 days	TS–related AIS was characterized by markedly elevated D-dimer, frequent adenocarcinoma, and multiterritorial DWI infarcts, with poor 90-day outcome (61.9%); this combination should prompt occult cancer screening in cryptogenic AIS
Oh MS [19]	2025	Prospective multicenter stroke-registry cohort linked to national claims data	9,759 AIS; 569 patients developed 976 occult cancer (5.8%)	Multiple occult cancer types	USE vs ISE	NA	NR	Median 4.4 years	Occult cancer incidence peaked immediately after AIS, particularly in USE (14.3 vs 7.6/1,000 person-months in ISE during month 1), declined rapidly, and stabilized after ≈12 months, supporting early cancer evaluation after unexplained AIS
Oka K [22]	2025	Single-center retrospective cohort	276 unresectable pancreatic/gastric cancer; 18 developed AIS	Unresectable pancreatic and gastric cancer	CAS	NR	NR	1-year incidence assessed; survival followed after stroke	All 18 AIS were classified as CAS. One-year incidence was 10.2% in pancreatic vs 5.8% in gastric cancer; post-stroke survival was shorter in pancreatic cancer (41 vs 116 days), although stroke itself did not significantly affect overall survival
Otani T [66]	2025	Retrospective autopsy-based comparative histopathological study	38 autopsy cases: 20 NBTE, 11 AF-related stroke, 7 IE-related stroke; 7 NBTE cerebral emboli available	16/20 NBTE had cancer; 13/20 advanced adenocarcinomas: lung 4, pancreatic 4, stomach 2, rectal 2, ovarian 1	NBTE-related cerebral embolism	Platelets (CD61), erythrocytes (glycophorin A), fibrin, vWF	NA	NA	NBTE vegetations and cerebral emboli were overwhelmingly platelet-dominant and erythrocyte-poor; this histological signature may identify NBTE as the source of retrieved cerebral thrombi
Chou SC [56]	2025	Experimental translational study (cell culture + murine pancreatic cancer model)	Pancreatic cancer cell lines + coagulation-deficient and orthotopic mouse models	Pancreatic cancer	NA	TF, FVIII, FIX, tumor-derived microvesicles	NA	NA	Thrombosis induced by TF-expressing pancreatic cancer microvesicles strongly depended on FVIII and FIX; vWF played a minor role. TF, but not FVIII, was significantly associated with tumor growth
Hyun JW [60]	2025	Single-center retrospective matched comparative study (biobank-based)	206 cancer + AIS: 50 plasma DNA, 40 nucleosomes, 56 cancer controls; 60 healthy controls	Multiple active cancers; hematologic malignancies excluded	Cancer + AIS; ESUS vs CSM subanalysis	Plasma cfDNA/DNA, nucleosomes, D-dimer	NR	NR	NETosis markers were higher in cancer + AIS than in cancer-only and healthy controls, correlated with D-dimer, and were particularly elevated in ESUS compared with CSM, supporting NETosis-related hypercoagulability
Woock M [67]	2025	Multicenter matched case–control study based on prospectively collected clot registry data (RESTORE)	128 LVO: 64 AC + 64 age/sex-matched non-cancer controls	Multiple cancers; colorectal 26.6%, GU 20.3%, lung/larynx 18.8%, pancreatic 10.9%; metastases 63%	LVO-AIS treated with MT: AC vs NAC	vWF, collagen; H3Cit exploratory	NA	NR	Cancer clots had higher vWF and collagen; H3Cit was higher before multiplicity correction. Platelet, fibrin, and RBC fractions did not significantly differ
Kim TJ [68]	2025	Prospective observational exploratory proteomic cohort	35 EVT treated AIS: CE 17, LAA 6, CAS 4, undetermined 8	Multiple active cancers	EVT-treated AIS: CE, LAA, cancer-related, undetermined	IGHG1 (CR); pleckstrin/PLEK (CE); CD59 (LAA)	NA	NR	Clot proteomics identified mechanism-specific protein signatures; IGHG1 characterized CAS, supporting thrombus proteomics as a potential tool for etiological classification
Oakley CI [76]	2025	Retrospective single-institution descriptive case series	112 Ca-NBTE; 92 stroke; 84 MRI available	Advanced adenocarcinoma 73.8%; lung most frequent, followed by pancreatic, GU, GI and hematologic	Ca-NBTE-related ischemic stroke	NR	Multifocal bihemispheric infarcts involving anterior/posterior circulations; Type IV disseminated infarcts 67.8%	NR	Ca-NBTE was most commonly associated with multifocal bihemispheric infarcts involving both anterior and posterior circulations. Disseminated infarcts of varying sizes predominated, although solitary/single-territory infarcts also occurred
Fu CH [103]	2025	Single-center prospective observational histopathological cohort	420 EVT-treated AIS: 50 AC, 23 NAC, 347 no cancer; prognostic analysis in 49 AC	Multiple cancers; adenocarcinoma 62%; lung and pancreatic most frequent	LVO-AIS treated with EVT; AC vs NAC vs no cancer	Fibrin/platelet proportion in retrieved thrombus	NA	90 days	Higher fibrin/platelet content in retrieved thrombi was associated with AC and independently predicted stroke recurrence or mortality within 90 days after EVT; FP ≥79.4% identified a high-risk phenotype
Chandu M [43]	2025	Single-center retrospective observational cohort	54 CAS; 41 completed follow-up	Hepatobiliary 18.5%, gynecological 18.5%, head/neck 16.6%, urogenital 13%, lung 11.1%, gastroesophageal 11.1%, hematologic 9.6%, breast 2%; metastases 25%	Undetermined/cryptogenic 62%; LAA 33.3%; CE 3.7%	D-dimer	Multi-vascular territory infarction 42%	3 months	Elevated D-dimer and multiterritorial infarction were characteristic of CAS; multiple vascular territory infarction clustered with hypercoagulability/metastatic disease and 3-month outcomes were poor (mRS 5–6 in 53%, mortality ≈44%)
Kim TJ [106]	2025	Retrospective nationwide linked registry–claims cohort	690	Stomach/esophagus 19.6%, colorectal 15.9%, lung 15.7%, hepatobiliary 15.1%, urological 11.7%, pancreatic 7.4%, others	CAS	NA	NA	Median 1.7 years (IQR 3 months–4.9 years)	Long-term prognosis was poor: 58.1% died, recurrent AIS occurred in 12.9% and major bleeding in 6.1%. Older age, greater stroke severity, anticoagulation at discharge, and pancreatic, hepatobiliary, and lung cancers independently predicted mortality
Kielkopf MC [92]	2025	Single-center retrospective cohort based on a prospective stroke registry	135 AC + AIS: 58 anticoagulant, 77 antiplatelet	Multiple; lung 22%, pancreatic 21%; metastases 48%	Undetermined 74.8%; ESUS 63.7%; LAA 17.8%; SVO 2.2%; other 5.2%; CE excluded	D-dimer	Multi-territory infarcts 30%	Median 495 days (IQR 57–1,029)	Anticoagulant and antiplatelet therapy showed similar adjusted risks of 1-year mortality and recurrent AIS; elevated D-dimer and multi-territory infarction independently predicted 1-year mortality
Xie L [80]	2025	Retrospective propensity score-matched case–control study	163: 41 CAS, 41 AIS without cancer, 40 cancer without stroke, 41 healthy controls	Adenocarcinoma 61%: respiratory 34.1%, digestive 29.3%, urinary 17.1%, endocrine 7.3%, female genital 7.3%, breast 4.9%	CAS vs non-cancer AIS	RBC count, D-dimer, 13-(S)-HODE	Multifocal cortical-subcortical infarction 75.6%; mixed large and small lesions 48.8%	NA	Lower RBC count, elevated D-dimer, and lower 13-(S)-HODE were independently associated with CAS; their combined model discriminated CAS from non-cancer AIS (AUC 0.819)
Ke X [105]	2025	Single-center retrospective cross-sectional observational study with survival follow-up	24 TS + AIS: 9 AIS-TS, 15 NAIS-TS	Multiple malignant tumors	TS related AIS	D-dimer index, CEA	Bilateral anterior + posterior circulation DWI infarction pattern	Median 30 days (IQR 26–46) AIS-TS; 86 days (IQR 56–393) NAIS-TS; follow-up ended Apr 5, 2024	AIS as the initial manifestation of TS was associated with worse survival. Female sex, elevated DDI, and elevated CEA independently predicted all-cause mortality
Gon Y [101]	2026	Prospective multicenter observational cohort (SCAN study)	135 AC + AIS; 84 with available TF activity data	Multiple cancers	AIS + AC; cryptogenic 57% (43% low-TF vs 71% high-TF)	TF activity, D-dimer, hs-CRP, vWF	Multiple infarcts: 61% high-TF vs 33% low-TF	1 year	Elevated plasma TF activity independently predicted 1-year mortality (HR 3.03, 95% CI 1.29–7.12) after adjustment for established clinical and biological prognostic factors; adenocarcinoma and distant metastasis associated with higher TF activity

*AIS* acute ischemic stroke, *NBTE* non-bacterial thrombotic endocarditis, *DIC* disseminated intravascular coagulation, *NR* not reported, *AC* active cancer, *CSM* conventional stroke mechanism, *DWI* diffusion-weighted imaging, *CAC* cancer-associated coagulopathy, *TCD* transcranial Doppler, *WT* wild type, *NA* not applicable, *PSGL-1* P-selectin glycoprotein ligand-1, *PAR4* protease-activated receptor 4, *CNS* central nervous system, *UCE* unidentified and/or cancer-associated stroke etiology, *CSE* conventional stroke etiology, *PWI* perfusion-weighted imaging, *MRA* magnetic resonance angiography, *CML* chronic myeloid leukemia, *NETs* neutrophil extracellular traps, *cfDNA* cell-free DNA, *G-CSF* granulocyte colony-stimulating factor, *CAS* cancer-associated stroke, *FDP* fibrin degradation products, *GI* gastrointestinal, *GU* genitourinary, *CE* cardioembolism, *LAA* large-artery atherosclerosis, *SVO* small-vessel occlusion, *HR* hazard ratio, *TOAST* Trial of Org 10,172 in Acute Stroke Treatment, *MRI* magnetic resonance imaging, *PPV* positive predictive value, *TIA* transient ischemic attack, *CRP* C-reactive protein, *SEER* Surveillance, Epidemiology, and End Results, *NAC* non-active cancer, *IQR* interquartile range, *OR* odds ratio, *AF* atrial fibrillation, *H3Cit* citrullinated histone H3, *hsTnT* high-sensitivity troponin T, *TAT* thrombin–antithrombin complex, *OASIS-CANCER* Optimal Anticoagulation Strategy In Stroke Related to CANCER, *EVs* extracellular vesicles, *CD326* cluster of differentiation 326/EpCAM, *EpCAM* epithelial cell adhesion molecule, *TF* tissue factor, *END* early neurologic deterioration, *aOR* adjusted odds ratio, *NIHSS* National Institutes of Health Stroke Scale, *NORSTROKE* Norwegian Stroke Research Registry, *Hb* hemoglobin, *CKD* chronic kidney disease, *LDL-C* low-density lipoprotein cholesterol, *VTE* venous thromboembolism, *AMBIs* acute multiple brain infarcts, *NLR* neutrophil-to-lymphocyte ratio, *TEACH* Trial of Enoxaparin versus Aspirin in Patients with Cancer and Ischemic Stroke, *DOAC* direct oral anticoagulant, *MT* mechanical thrombectomy, *CD42b* cluster of differentiation 42b/glycoprotein Ib alpha platelet marker, *DNA* deoxyribonucleic acid, *TTS* three-territory sign, *TS* Trousseau syndrome, *SUE* stroke of undetermined etiology, *SOE* stroke of other determined etiology, *EVT* endovascular therapy, *RBC* red blood cell, *WBC* white blood cell, *AUC* area under the curve, *OSM* occult systemic malignancy, *AUROC* area under the receiver-operating characteristic curve, *SAO* small-artery occlusion, *CEA* carcinoembryonic antigen, *CA125/CA-125* cancer antigen 125, *CA19-9* carbohydrate antigen 19–9, *sICAM-1* soluble intercellular adhesion molecule−1, *sVCAM-1* soluble vascular cell adhesion molecule−1, *SVD* small-vessel disease, *NSCLC* non-small cell lung cancer, *ASTRAL* Acute STroke Registry and Analysis of Lausanne, *HCC* hepatocellular carcinoma, *HCRCI* hepatocellular carcinoma-related cerebral infarction, *TEG* thromboelastography, *MA* maximum amplitude, *AFP* alpha-fetoprotein, *ATE* arterial thromboembolism, *KRAS* KRAS proto-oncogene, *STK11* serine/threonine kinase 11, *SVS* susceptibility vessel sign, *HVS* hyperdense vessel sign, *CT* computed tomography, *SCAN* SCAN study, *DVT* deep-vein thrombosis, *PE* pulmonary embolism, *ESR* erythrocyte sedimentation rate, *aPTT* activated partial thromboplastin time, *PT* prothrombin time, *INR* international normalized ratio, *PLR* platelet-to-lymphocyte ratio, *ICAM-1* intercellular adhesion molecule−1, *LVO* large-vessel occlusion, *NPV* negative predictive value, *CAH* cancer-associated hypercoagulability, *ENCHASE* Edoxaban for the Treatment of Hypercoagulability in Patients with Active Cancer and Acute Ischemic Stroke, *ESUS* embolic stroke of undetermined source, *RCT* randomized-controlled trial, *ARCADIA* Atrial Cardiopathy and Antithrombotic Drugs in Prevention After Cryptogenic Stroke, *CI* confidence interval, *vWF* von Willebrand factor, *mRS* modified Rankin Scale, *USE* unidentified stroke etiology, *ISE* identified stroke etiology, *IE* infective endocarditis, *CD61* cluster of differentiation 61/glycoprotein IIIa platelet marker, *FVIII* coagulation factor VIII, *FIX* coagulation factor IX, *RESTORE* registry of retrieved thrombotic material from large-vessel occlusion stroke, *IGHG1* immunoglobulin heavy constant gamma 1, *CR* cancer-related, *PLEK* pleckstrin, *CD59* cluster of differentiation 59, *Ca-NBTE* cancer-associated non-bacterial thrombotic endocarditis, *FP* fibrin/platelet, *13-(S)-HODE* 13(S)-hydroxyoctadecadienoic acid, *AIS-TS* acute ischemic stroke as the initial manifestation of Trousseau syndrome *NAIS-TS* acute ischemic stroke not presenting as the initial manifestation of Trousseau syndrome, *DDI* D-dimer index

### Epidemiology

Active cancer is present in 5%–10% of patients [13] with ischemic stroke, particularly among those with embolic ischemic stroke of undetermined origin (ESUS) [14, 15], whether the malignancy has been previously diagnosed or not [7, 15, 16].

In 3% to 5% of these patients, stroke precedes the diagnosis of an occult malignancy by up to 2 years [17], constituting the first clinical manifestation of cancer [18]. Consistently, occult malignancy is identified in 5.3% of patients with ESUS, with the highest detection rate occurring in the early period following stroke (14.3 per 1000 person‑months) [19].

In this context, arterial thromboembolism (ATE) shows a characteristic temporal pattern, with a peak incidence within the first year after cancer diagnosis, and stroke accounts for the majority of ATE events (71.6%) [20].

Among patients with cancer, the most frequent cause of stroke is CAC (39.8%), whereas conventional stroke mechanisms are less common [17, 21–24]. Similarly, traditional cardiovascular risk factors are less prevalent in cancer patients than in stroke patients without cancer [25, 26].

Temporally, the risk of stroke related to hypercoagulability is highest during the first six months after cancer diagnosis, and many patients have already reached the metastatic stage at the time of stroke presentation [7, 8, 16, 26–30]. Although this risk declines after the first year, it remains higher than in the general population even up to a decade later [31].

Although all tumor types may be associated with CAC-related stroke, adenocarcinoma is the histological subtype most frequently implicated [8, 19, 24, 26, 27, 29, 30, 32–36]. The cumulative incidence rate of stroke due to hypercoagulability is highest for lung cancer, followed by pancreatic, colorectal, breast, and prostate cancer [7, 26, 37]. Notably, the interval between cancer diagnosis and stroke is particularly short in pancreatic and colorectal cancer, underscoring their strong thrombotic potential [38, 39].

### Clinical manifestations

Patients with cryptogenic stroke and active cancer typically present with a lower body mass index, poorer nutritional status, younger age, female sex, fewer atherosclerotic risk factors, and metastatic disease [7, 24, 40].

In addition, CAC-related strokes are usually more severe and have a poor clinical course, with rapid neurological deterioration, progressive worsening, or early recurrences [24, 30, 35, 41–43].

**Fig. 2:**
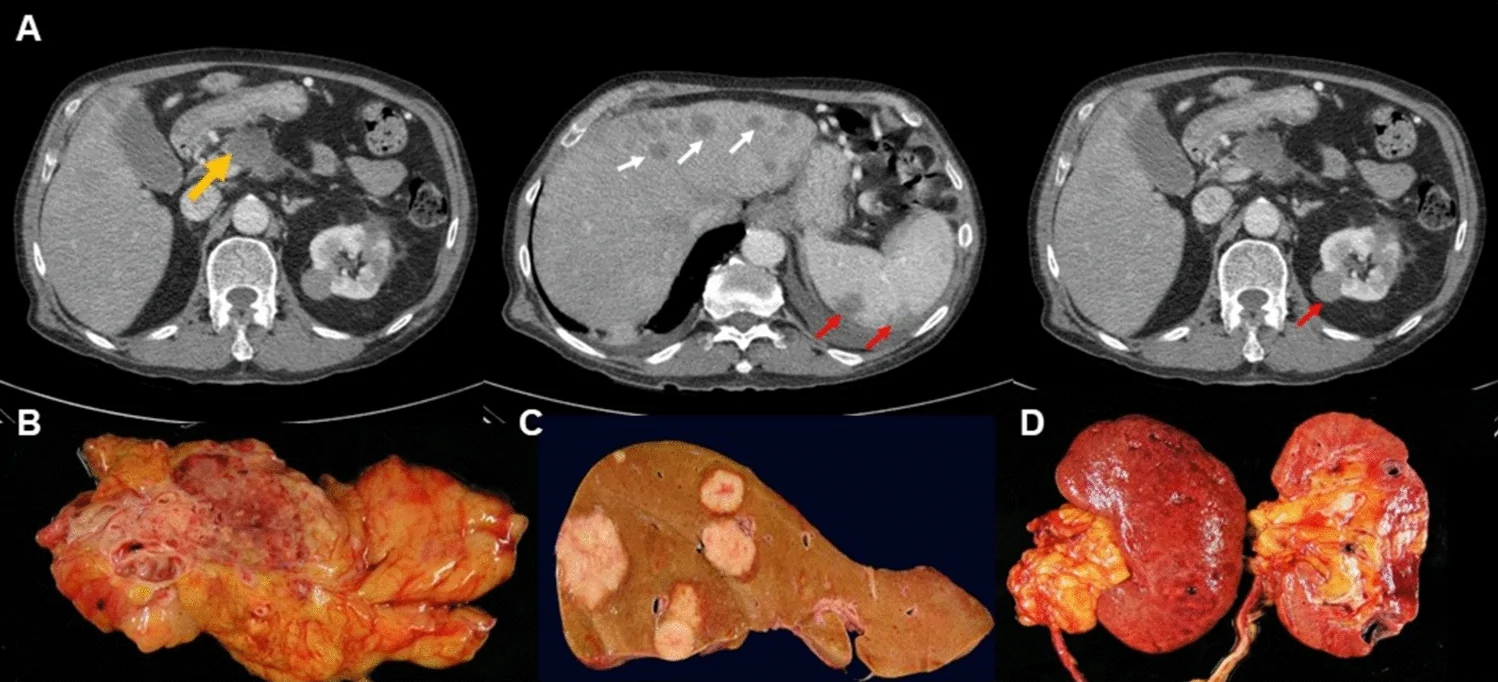
**A** Abdominal CT scan showing a pancreatic lesion compatible with a tumor (orange arrow), together with liver metastases (white arrows) and splenic and renal infarcts (red arrows). **B-D** Macroscopic autopsy photographs of the same patient showing the pancreatic tumor (A), the liver with metastases (B) and the kidney with areas of infarction

In contrast to infarctions associated with hypercoagulability in other organs, such as the spleen or kidney, which often remain subclinical and are detected only on imaging (Fig. 2 A, B),  CAC-related cerebral infarctions are commonly symptomatic and frequently multiple [21, 24].

**Fig. 3:**
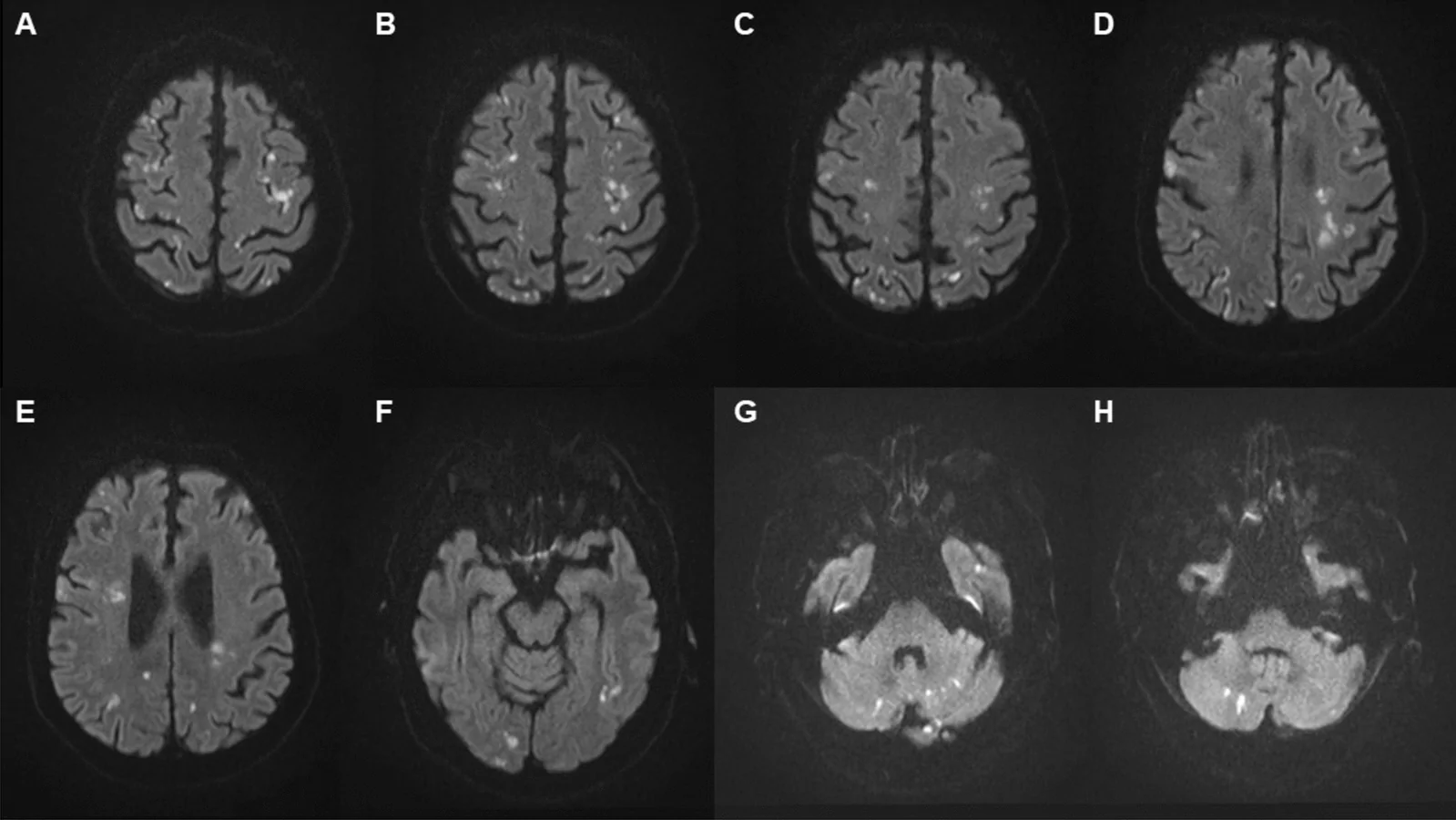
A-E. Diffusion-weighted axial cranial magnetic resonance images, **A-E** multiple bihemispheric cortical lesions affecting the anterior circulation; **F** lesions in the posterior circulation; **G, H** cerebellar involvement

Up to 50% of patients with active cancer experience simultaneous ischemic strokes, compared to 10% of patients without cancer [25, 44]. In some cases, these patients may develop encephalopathy [45], resulting from small cortical ischemic lesions in multiple vascular territories **(**Fig. 3**)** [41].

### Pathogenesis of cancer-associated coagulopathy

Trousseau's syndrome constitutes a spectrum of disorders defining a state of hypercoagulability associated with any type of malignancy and includes venous thromboembolism (VTE), ATE (stroke or myocardial infarction), disseminated intravascular coagulation (DIC), and non-bacterial or marantic thrombotic endocarditis (NBTE) [46].

**Fig. 4:**
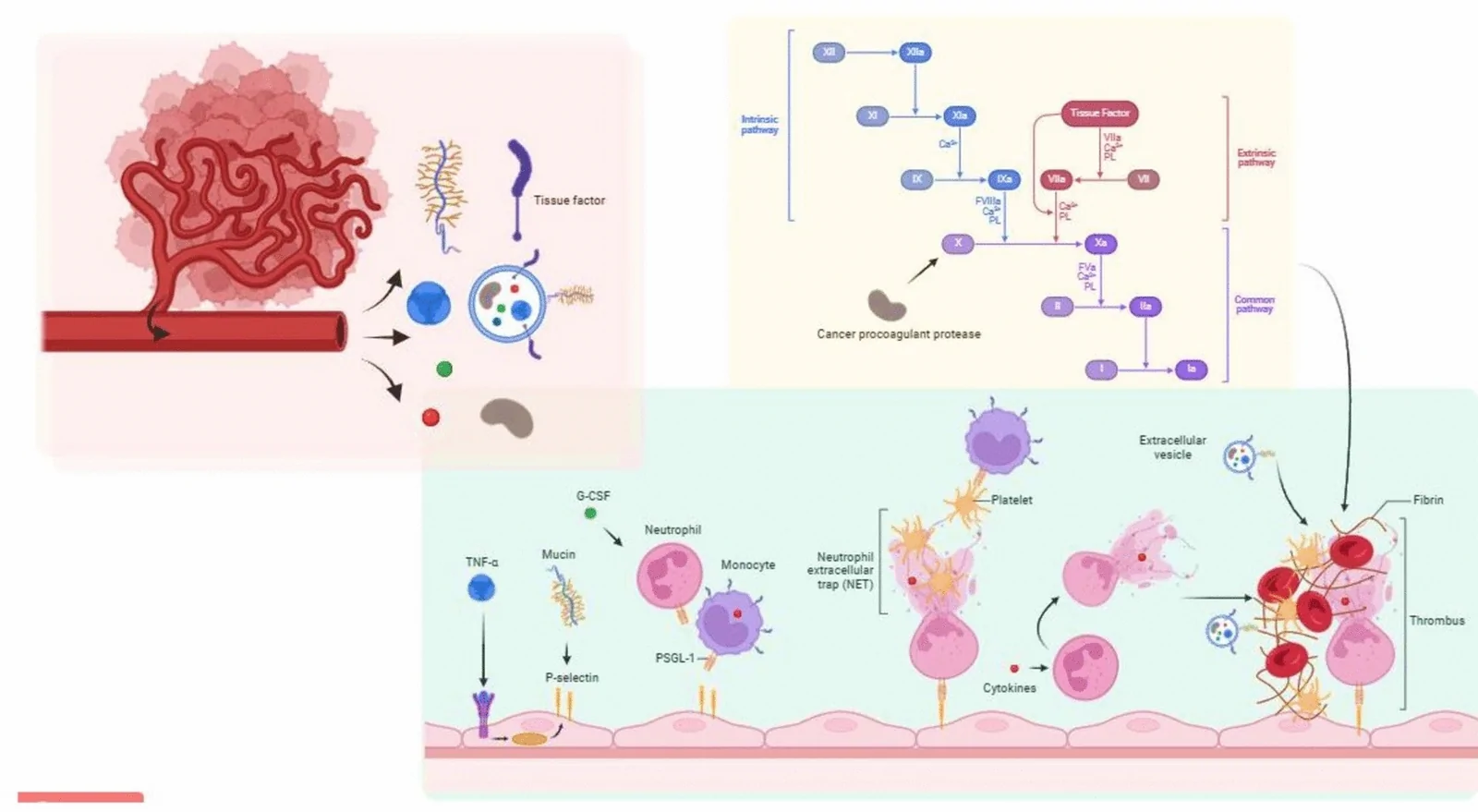
Schematic diagram summarizing the pathophysiological mechanisms of cancer-related coagulopathy

This hypercoagulable state is the most frequent mechanism underlying cancer-related stroke [2, 15, 16, 21–24, 30, 47]. Multiple overlapping and interacting pathophysiological mechanisms have been proposed to explain the cancer-induced hypercoagulable state (Fig. 4), which is ultimately mediated by cancer-related inflammation, endothelial dysfunction, platelet activation, and circulating microemboli [48–50].

On the one hand, adenocarcinomas produce mucin, a highly glycosylated molecule that, although largely cleared by the liver, can interact with adhesion molecules (P-selectin and L-selectin), inducing the formation of platelet-rich microthrombi [51–53].

In addition, it has long been known that tumor cells overexpress tissue factor (TF) [54], a transmembrane receptor protein, which plays a crucial role in activating the coagulation cascade via the extrinsic pathway. This expression is up-regulated by oncogenic events such as inactivation of the *p53* tumor suppressor gene and activating mutations in the *K-ras* oncogene [54]. More recently, alterations in *K-ras* and the *STK11* tumor suppressor gene have been associated with an increased risk of ATE [55]. Regarding stroke, the HR among individuals with vs without *K-ras* alterations was 2.22 (95% CI: 1.38–3.58), compared with 3.48 (95% CI: 1.87–6.46) among individuals with vs without *STK11* alterations [55].

A very recent in vitro study in human pancreatic cancer cell lines demonstrated that thrombosis induced by TF-expressing microvesicles depends predominantly on coagulation factors VIII and IX, whereas von Willebrand factor (vWF) plays a minor role [56].

Studies on circulating extracellular vesicles (EVs) secreted from the tumoral cells have contributed to understanding cancer-associated thrombosis. Serum levels of EVs expressing different cancer cell markers have been measured using flow cytometry [57, 58]. The levels of cancer cell-derived EVs were higher in cancer-related stroke patients than in other groups and correlated with D-dimer levels, but not with EV-associated TF, supporting the existence of additional prothrombotic mechanisms beyond the TF-dependent pathway [57]. In vitro experiments investigating the ability of cancer cell-derived EVs from different cell lines to induce clot formation concluded that EVs from adenocarcinoma were associated with shorter clotting times compared with those from squamous cell carcinoma [58].

Another mediator of cancer‑associated thrombosis is neutrophil extracellular trap formation (NETosis) [59]. Using solid tumor animal models, *Demers *et al*.* showed that cancers can induce an increase in peripheral blood neutrophils that are sensitized toward NETosis by granulocyte colony-stimulating factor (G-CSF), and that extracellular chromatin released through NETosis may contribute to cancer-associated thrombosis. In cancer patients with stroke, levels of NET-specific biomarkers, such as plasma DNA, nucleosome, and citrullinated histone H3 (H3Cit), are significantly elevated, and these markers correlate with D-dimer levels and thrombin–antithrombin complex, indicating NET-associated coagulopathy [60–62]. Furthermore, a post-mortem histopathological study of ischemic stroke patients revealed disseminated, widespread microvascular thrombosis, with immunohistochemistry demonstrating the presence H3Cit-positive cells in multiple cerebral thrombi [62].

Thrombi extracted by thrombectomy in patients with acute stroke have provided insights into the pathogenesis of CAC. Thrombi retrieved from stroke patients with active cancer are platelet-rich and erythrocyte-poor, which is more evident in patients with NBTE [63–66]. A recent study analyzing thrombi using immunohistochemical techniques found that clots from patients with concomitant cancer had a significantly higher content of vWF and H3Cit [67], indicating a cancer- driven effect on primary hemostasis.

Finally, using proteomic analysis, potential clot protein biomarkers linked to cancer-related stroke mechanisms have been identified. Cancer-related clots exhibited high levels of proteins associated with active cancer and immune responses, such as IGHG1 and VTN [68].

### Radiological characteristics

The most characteristic radiological pattern of stroke secondary to CAC consists of multiple ischemic lesions spanning more than two vascular territories on diffusion-weighted imaging (DWI) in magnetic resonance imaging (MRI) [7, 25, 26, 33, 40, 41, 69, 70]. In addition, microembolic dispersion of infarcts is found in 78% of these patients, and 50% have previous infarcts [41].

The number of affected arterial territories on DWI-MRI serves as an independent predictor of occult malignancy in patients with cryptogenic stroke [69]. In line with this, *Finelli *et al. described the “three territories sign” as a diagnostic imaging feature to identify cerebral infarcts associated with malignancy [71]. This sign is defined by the presence of multiple lesions on DWI-MRI affecting three vascular territories, including both anterior and posterior circulation, simultaneously and bilaterally **(**Fig. 3A-F**)**. In the absence of infective endocarditis, this sign is a highly specific marker of CAC-related stroke [29, 71–76]. In studies of patients with three-territory infarcts, approximately 75% of cases without an identifiable embolic source are malignancy-related [71].

The most frequently affected brain areas are the cortical and subcortical regions, followed by the cerebellar hemisphere and the corpus callosum, while deep brain structures and the brainstem are rarely affected [26, 71].

The absence of the susceptibility vessel sign (SVS) on susceptibility-weighted imaging (SWI) in patients with large vessel arterial occlusion has been associated with active cancer, with a reported sensitivity of 27% and specificity of 85% [77], and may help identify paraneoplastic hypercoagulability in stroke patients with active malignancy [78]. This finding is thought to be driven by differences in thrombus composition, characterized by a higher proportion of fibrin and platelets, and a lower erythrocyte content [63].

### Biomarkers

Currently, there are no clear and validated algorithms available to identify patients with occult neoplasms among those with cryptogenic stroke. It is not cost-effective to perform tumor screening tests in all these patients [18]. However, several studies have suggested the inclusion of specific biomarkers in routine clinical practice.

D-dimer, although non-specific, has been consistently associated with CAC and with elevated fibrin degradation products in cancer-associated ischemic stroke [18, 26, 34, 40, 70, 74, 75, 79, 80]. *Guo *et al. proposed a D-dimer cut-off at or above 5.5 μg/ml for cancer-associated stroke, with a specificity of 99.7% and a positive predictive value (PPV) of 92.9%, regardless of imaging characteristics [75], whereas *Wang JY *et al. later suggested a lower threshold of 2.785 μg/ml, with a sensitivity of 50.9% and a specificity of 98.5% [74]. Beyond its diagnostic value, higher D-dimer concentrations have been reported in patients with active cancer and stroke, who more frequently exhibit adenocarcinoma histology and systemic metastases [34].

*Seok *et al*.* demonstrated a significant correlation between D-dimer levels and the number of microembolic signals detected by transcranial Doppler (TCD) in patients with CAC [81]. In a prospective study, *Navi *et al. evaluated coagulation factors, platelet activation markers, endothelial activation markers, and TCD-detected microembolic signals in three groups of patients: those with cancer and stroke, those with cancer alone, and those with stroke without cancer. The results showed that patients with cancer and stroke who had elevated levels of D-dimer, P-selectin, sICAM-1, sVCAM-1, and TCD-detected microembolic signals were at increased risk of thromboembolic events or death. In contrast, thrombin–antithrombin complex and thrombomodulin levels were not associated with thromboembolism or mortality [48]. To date, D-dimer remains the only biomarker consistently associated with recurrent stroke in this population [48, 82–84].

*Cocho *et al. found that in patients with cryptogenic stroke, C-reactive protein (CRP) levels above 20 mg/l have a sensitivity of 75% and a specificity of 96%, and that fibrinogen levels above 600 mg/dL have a sensitivity of 67% and a specificity of 96% for predicting occult malignancy [17]. Subsequent studies showed that patients with stroke and active cancer have lower hemoglobin and hematocrit levels, and higher fibrinogen and CRP levels compared to patients with stroke and cancer in remission [17, 36, 38, 80]. In this context, elevated D-dimer and CRP levels further support a strong association between hypercoagulability and stroke in cancer-related strokes [39].

The level of CA-125, a tumor marker associated with mucin-producing neoplasms, may be a useful biomarker, as it has been shown to be elevated in patients with stroke secondary to CAC and correlates with D-dimer levels [29, 85].

*Kawano *et al. reported that an elevated neutrophil-to-lymphocyte ratio (NLR) greater than 15 at the time of cancer diagnosis identifies patients at high risk for ischemic stroke within the following two years [86, 87]. Similarly, higher neutrophil counts, along with elevated D-dimer and α-fetoprotein levels, have been independently associated with cancer-related stroke [23].

The NORSTROKE study developed a score to predict occult malignancy [18]. Based on this scale, if a patient under 75 years of age with ischemic stroke of undetermined etiology has D-dimer levels above 3 mg/L, hemoglobin less than 12 g/dL, and is or has been a smoker, the probability of having an active cancer is 53%.

### Treatment and prevention

While there are numerous guidelines and recommendations on prevention and treatment of VTE [88], the same cannot be said for ATE, and specifically for the treatment of stroke caused by CAC. To choose secondary prevention of cancer-related stroke, it is necessary to assess bleeding risk and anticoagulation indication based on the presence of atrial fibrillation, NBTE, or VTE.

If anticoagulation is indicated, low-molecular-weight heparin (LMWH) or direct-acting anticoagulants (DOACs) are commonly used for secondary prevention of stroke related to cancer-associated hypercoagulability, whereas LMWH is generally preferred in patients with gastrointestinal malignancies because of the higher bleeding risk associated with DOACs. Vitamin K antagonists (VKAs) are less effective in this setting, although they may be considered when LMWH and DOACs are contraindicated [89–91].

In a recent retrospective study, no differences in mortality or recurrences were found in patients with stroke and active cancer treated with anticoagulant therapy versus antiplatelet therapy [92]. Anticoagulants were more frequently chosen in younger patients, with elevated D-dimer and advanced cancer.

Effective correction of hypercoagulability may therefore contribute to improved survival in patients with active cancer and stroke [20, 34].

Comparative studies between LMWH and DOACs have shown broadly similar clinical outcomes. DOACs demonstrated comparable efficacy and safety to LMWH in cryptogenic ischemic stroke in patients with active cancer, and no significant differences were observed in major bleeding, thromboembolic events, or survival when comparing enoxaparin with aspirin, DOACs with enoxaparin, or DOACs with warfarin [90, 93, 94]. Likewise, oral edoxaban performed similarly to subcutaneous enoxaparin in CAC [95], and major ischemic or hemorrhagic events did not differ significantly between apixaban and aspirin in patients with a history of cancer and cryptogenic stroke [96].

Additionally, recent scientific statement emphasizes the importance of conducting the necessary studies to identify the specific cause of stroke in cancer patients [97]. In those patients with CAC-related stroke, the authors conclude that there is insufficient evidence to choose between anticoagulation and antiplatelet therapy [97].

Taking into account most of the studies we have reviewed here, *Alizou *et al. published a proposal of treatment algorithm [98]. If anticoagulation is indicated and bleeding risk is high (in case of gastrointestinal tumors), LMWH is preferred. When there is not high bleeding risk, LMWH and DOACs may be both indicated. For patients without clear anticoagulation indications, aspirin is recommended.

Regarding treatment duration, current therapeutic guidelines for cancer-associated thrombosis recommend maintaining anticoagulation for at least 3–6 months, with extended therapy advised when cancer remains active or thrombotic risk is high.

### Prognosis

In general terms, CAC-related stroke has a poor prognosis [24, 33, 34]. The median modified Rankin Scale (mRS) score at hospital discharge is 3, and at 3 months, 75.6% of patients have an mRS score ≥ 3 [43, 99]. Functional outcomes remain poor despite acute stroke treatment; approximately half of patients die within three months, and 50% of survivors at 3 months continue to experience significant disability [16, 99, 100].

Prognosis is even worse when stroke is the first manifestation of an occult malignancy, with a median survival of only 58 days [16]. Moreover, cryptogenic stroke in the context of active cancer is independently associated with higher in-hospital mortality and reduced overall survival [24, 26, 27, 36]. In addition, elevated TF activity has been independently associated with 1-year mortality in patients with active cancer and stroke [101].

Recurrent stroke is also more frequent in this population. Patients with cancer have a recurrence rate of 13.6%, approximately three times higher than those without cancer, with cumulative rates of 7% at 1 month, 13% at 3 months, and 16% at 6 months [42, 99]. D-dimer levels are consistently associated with recurrent stroke and mortality, and high plasma D-dimer concentrations after anticoagulant therapy predict poor short-term outcomes and early recurrence in cancer-associated stroke [35, 48, 83, 84]. Additional biomarkers, such as elevated vWF antigen levels, have also been linked to adverse outcomes, [102] and in patients undergoing endovascular treatment, a fibrin platelet proportion ≥79.4% in retrieved thrombi predicts stroke recurrence or mortality at 90 days [103].

The 30-day mortality rate for CAC-related stroke ranges from 25 to 50%, compared to 14% in non-cancer patients [43, 46], and more than half of deaths occur within 6 months of stroke [1]. The median survival in cancer patients with cryptogenic stroke ranges from 62 to 365 days [89, 104], and is significantly shorter than in patients with stroke of other causes (34 vs. 590 days) [100]. The high D-dimer levels, systemic metastases, and diabetes are independent clinical predictors of poor survival [35, 38, 89, 105], and in patients with NBTE, up to 90% die or experience recurrent stroke within 6 months [47]. Other factors associated with long-term mortality have included female sex, elevated carcinoembryonic antigen levels [105], old age, anticoagulation therapy at discharge, and pancreatic, lung, and hepatobiliary tumors [106].

## Discussion

Approximately 40% of ischemic strokes in patients with active cancer may be attributable to cancer-specific pathophysiological mechanisms leading to a hypercoagulable state [7, 25].

The histological type of cancer most frequently associated with CAC-related stroke is adenocarcinoma, and the malignancies most frequently associated with this entity are lung, pancreatic, colorectal, breast, and prostate cancers [37]. Advanced and metastatic stages of cancer are associated with an increased risk of stroke due to hypercoagulability.

The pathophysiological mechanisms involved in CAC are diverse and promote the formation of platelet-rich microthrombi. These mechanisms include mucin production, release of proinflammatory cytokines, TF-rich microparticles derived from malignant cells, circulating extracellular vesicles secreted by tumor cells, and neutrophil extracellular trap formation.

Brain MRI can identify characteristic patterns of CAC-related stroke, consisting of multiple ischemic lesions affecting more than two vascular territories [41]. The most specific radiological finding is known as the “three territories sign” [71].

D-dimer, although non-specific, is currently the most useful biomarker for identifying CAC and predicting the risk of recurrent stroke [83]. Interpretation of D-dimer thresholds should be undertaken cautiously because studies used different analytical platforms and reported concentrations using either fibrinogen-equivalent units (FEU) or D-dimer units (DDU), which limits direct comparison across studies and precludes establishment of a universal diagnostic cutoff. Other potential biomarkers include the number of microembolic signals detected by TCD, CRP, fibrinogen, prothrombin time, hemoglobin, hematocrit, and CA-125, all of which demonstrate limited specificity and sensitivity [17, 48, 81, 85].

The main clinical and radiological characteristics of patients with ischemic stroke secondary to CAC are summarized in Table 2.

**Table 2:** Clinical-radiological characteristics of patients with stroke due to cancer-associated coagulopathy

Stroke	Data	Cancer	Data
Encephalopathy secondary to multi-territorial strokes	29.4% [81]	Mucin-producing adenocarcinoma	68%[7, 27]; 81%
First manifestation of cancer	1.5%^a^ [24]	Recent diagnosis	1.29%^a^[20]
Early recurrences	18% despite ACo [83]; 16% in ACp [30]	Lung/pancreas/GI	76.4% [7] Lung 27%, pancreas 12%, GI 25.9 (CRC 8.5%) [35]
Indeterminate or cryptogenic	51% in ACp [27]	Advanced or metastatic stage	70% [27, 29]
Less common classical vascular risk factors	DLP 23%, AF 20.6%, HT 63%^c^ [36]	Constitutional syndrome (BMI)	19.7 (16.9–22.4)^e^ [7]
TCD-detected microembolic signals	58% [27, 81]	D-dimer elevation	10.1 µg/ml (5–27.3)^e^[29]
Sign of the three territories: multiple lesions on cranial MRI-DWI	84% [41]	Silent infarcts in other organs (spleen, kidney)	16.6% [76]
Poor functional prognosis 50%^d^ [43, 99]			

*ACp* active cancer patients, *ACo* anticoagulation, *AF* atrial fibrillation, *AIS* acute ischemic stroke, *BMI* body mass index, *CRC* colorectal cancer, *DLP* dyslipidemia, *HT* hypertension, *GI* gastrointestinal cancer, *MRI-DWI* magnetic resonance diffusion imaging, *TCD* transcranial Doppler

^a^Overall incidence of newly diagnosed cancer in the year after stroke

^b^Cumulative stroke incidence during the first year after cancer diagnosis (accounting for more than one-half of the overall 5-year incidence rate − 2.32%-)

^c^Non active cancer patients DLP 42.7%, AF 37.4%, HT 82.4% *P* value < 0.001

^d^3-month survivors despite acute stroke treatment

^e^median and interquartile range

The recommended secondary prevention treatment for CAC-related stroke is LMWH or DOACs. However, despite treatment, prognosis remains poor. More than 75% of patients are at least moderately disabled three months after stroke [99], the risk of recurrence is approximately threefold higher than in patients without cancer [30], and up to 50% of cancer patients die within 30 days after stroke [46]. These findings highlight the challenges associated with the management of CAC-related stroke and underscore the need for more effective and personalized antithrombotic strategies to improve clinical outcomes.

The main limitation of this review is the predominance of retrospective and observational studies among the included evidence, together with the limited availability of randomized clinical trials evaluating diagnostic approaches or treatment strategies in CAC-related stroke. In addition, the review protocol was not prospectively registered in PROSPERO, which may have increased the risk of reporting bias. Equally, a quantitative meta-analysis was not performed because of marked heterogeneity regarding studies design, patient populations, definitions of CAC, D-dimer assays and units, biomarker thresholds, outcome measures, and follow-up periods. Finally, only studies published in English or Spanish were included, which may have introduced language bias and limited identification of relevant studies published in other languages.

Nevertheless, this review has several strengths. It provides a comprehensive and up-to-date synthesis of the available evidence on a clinically relevant and increasingly recognized cause of ischemic stroke, addressing it from a broad perspective that ranges from epidemiology to clinical manifestations, including pathogenic mechanisms and therapeutic approaches.

Further research is needed to improve the understanding of CAC-related stroke pathophysiology. Since this is a rare cause of stroke, prospective multicentre cohort studies are also needed to enable the recruitment of large cohorts of patients. It is also necessary to validate reliable diagnostic and prognostic biomarkers, standardize D-dimer assays, and perform external validation of NORSTROKE. Finally, it is important to perform randomized trials comparing LMWH vs DOAC vs antiplatelet therapy in order to develop personalized antithrombotic strategies aimed at reducing recurrence and improving clinical outcomes in CAC-related stroke.

## Conclusion

CAC-related stroke is a distinct and underrecognized entity characterized by specific clinical, biological and radiological features and poor outcomes. Given the expected worldwide increase in cancer incidence and improvements in cancer survival, the burden of CAC-related stroke is likely to rise substantially in the coming years, highlighting the importance of improving its recognition and management.

## Supplementary Information

Below is the link to the electronic supplementary material.

Supplementary file1 (DOCX 18 kb)

Supplementary file2 (DOCX 150 kb)

Supplementary file3 (DOCX 156 kb)

## Data Availability

No new datasets were generated. All data analyzed in this study were derived from previously published articles.
