# Supplementary material for: Stroke secondary to cancer-associated coagulopathy: a systematic review
Source: J Neurol. 2026 Sep 20;273(10):609. doi: 10.1007/s00415-026-14119-y (PMC13590450; doi:10.1007/s00415-026-14119-y)
Supplement: Supplementary file 1 [file 415_2026_14119_MOESM1_ESM.docx]

**STROKE SECONDARY TO CANCER-ASSOCIATED COAGULOPATHY: A SYSTEMATIC REVIEW.**

Jauregui Larrañaga C^1,2#^, Gómez Arteta E^3^, Marta Enguita J^4,5*^, Erro Aguirre ME^6,7*^

^1^Neurology Department. Hospital Universitario Donostia. Donostia-San Sebastián.

^2^Biogipuzkoa Health Research Institute. Donostia-San Sebastián.

^3^Hematology Department. Hospital Universitario de Navarra. Pamplona.

^4^Neurology Department. Hospital Universitario Marqués de Valdecilla. Santander.

^5^Neurological Clinical Research Group, Instituto Investigación Sanitaria Valdecilla (IDIVAL), Santander.

^6^Neurology Department. Hospital Universitario de Navarra (HUN). Pamplona.

^7^Neuroepigenetics Laboratory-Navarrabiomed-Universidad Pública de Navarra (UPNA)-Instituto de investigación sanitaria de Navarra (IdiSNA), Pamplona.

*These authors share senior co-authorship.

Article type: Systematic review

(#) author of correspondence:

Carlota Jauregui Larrañaga, MD

Neurology Department.

Hospital Universitario Donostia

Begiristain Doktorea Pasealekua, 20014 Donostia (Gipuzkoa)

Tel.: +943 007000

e-mail: carlotajaureguilarranaga@gmail.com

https://orcid.org/**0000-0002-7963-5169**

**Supplementary Table 1.** Full Boolean queries used for each database

|  | **PubMed/MEDLINE** | **Scopus** | **Cochrane Library** | **Embase (Elsevier)** |
| --- | --- | --- | --- | --- |
| Full Boolean query | (("Stroke"[Mesh] OR "Brain Ischemia"[Mesh] OR "Cerebral Infarction"[Mesh] OR "ischemic stroke"[tiab] OR "ischaemic stroke"[tiab] OR "cerebral infarct*"[tiab] OR "brain ischemi*"[tiab]) AND ("Neoplasms"[Mesh] OR cancer*[tiab] OR neoplasm*[tiab] OR malignan*[tiab] OR tumor*[tiab] OR tumour*[tiab] OR carcinoma*[tiab] OR adenocarcinoma*[tiab]) AND ("Blood Coagulation Disorders"[Mesh] OR coagulopath*[tiab] OR hypercoagulab*[tiab] OR thrombophili*[tiab] OR "Trousseau syndrome"[tiab] OR "disseminated intravascular coagulation"[tiab] OR "nonbacterial thrombotic endocarditis"[tiab] OR "non-bacterial thrombotic endocarditis"[tiab] OR "marantic endocarditis"[tiab]) ) OR ("cancer-associated stroke"[tiab] OR "cancer associated stroke"[tiab] OR "cancer-related stroke"[tiab] OR "cancer related stroke"[tiab] OR "cancer-associated ischemic stroke"[tiab] OR "cancer-associated ischaemic stroke"[tiab] OR "cancer-associated hypercoagulation"[tiab] OR "cancer-associated hypercoagulability"[tiab] OR "cancer-associated coagulopathy"[tiab] OR "Trousseau syndrome"[tiab] AND (stroke[tiab] OR infarct*[tiab])) | TITLE-ABS-KEY( (("ischemic stroke" OR "ischaemic stroke" OR "cerebral infarct*" OR "brain ischemi*") AND (cancer* OR neoplasm* OR malignan* OR tumor* OR tumour* OR carcinoma* OR adenocarcinoma*) AND (coagulopath* OR hypercoagulab* OR thrombophili* OR "Trousseau syndrome" OR "disseminated intravascular coagulation" OR "nonbacterial thrombotic endocarditis" OR "non-bacterial thrombotic endocarditis" OR "marantic endocarditis") ) OR "cancer-associated stroke" OR "cancer associated stroke" OR "cancer-related stroke" OR "cancer related stroke" OR "cancer-associated ischemic stroke" OR "cancer-associated ischaemic stroke" OR "cancer-associated hypercoagulation" OR "cancer-associated hypercoagulability" OR "cancer-associated coagulopathy" OR ("Trousseau syndrome" AND (stroke OR infarct*)) | #1 MeSH descriptor: [Stroke] explode all trees #2 MeSH descriptor: [Brain Ischemia] explode all trees #3 MeSH descriptor: [Cerebral Infarction] explode all trees #4 ("ischemic stroke" OR "ischaemic stroke" OR "cerebral infarct*" OR "brain ischemi*"):ti,ab,kw #5 #1 OR #2 OR #3 OR #4  #6 MeSH descriptor: [Neoplasms] explode all trees #7 (cancer* OR neoplasm* OR malignan* OR tumor* OR tumour* OR carcinoma* OR adenocarcinoma*):ti,ab,kw #8 #6 OR #7  #9 MeSH descriptor: [Blood Coagulation Disorders] explode all trees #10 (coagulopath* OR hypercoagulab* OR thrombophili* OR "Trousseau syndrome" OR "disseminated intravascular coagulation" OR "nonbacterial thrombotic endocarditis" OR "non-bacterial thrombotic endocarditis" OR "marantic endocarditis"):ti,ab,kw #11 #9 OR #10  #12 (#5 AND #8 AND #11) #13 ("cancer-associated stroke" OR "cancer associated stroke" OR "cancer-related stroke" OR "cancer related stroke" OR "cancer-associated ischemic stroke" OR "cancer-associated ischaemic stroke" OR "cancer-associated hypercoagulation" OR "cancer-associated hypercoagulability" OR "cancer-associated coagulopathy"):ti,ab,kw #14 ("Trousseau syndrome":ti,ab,kw AND (stroke OR infarct*):ti,ab,kw) #15 #12 OR #13 OR #14 | (('ischemic stroke'/exp OR 'brain ischemia'/exp OR 'brain infarction'/exp OR 'ischemic stroke':ti,ab,kw OR 'ischaemic stroke':ti,ab,kw OR 'cerebral infarct*':ti,ab,kw OR 'brain ischemi*':ti,ab,kw) AND ('neoplasm'/exp OR cancer*:ti,ab,kw OR neoplasm*:ti,ab,kw OR malignan*:ti,ab,kw OR tumor*:ti,ab,kw OR tumour*:ti,ab,kw OR carcinoma*:ti,ab,kw OR adenocarcinoma*:ti,ab,kw) AND ('blood clotting disorder'/exp OR 'hypercoagulability'/exp OR 'disseminated intravascular coagulation'/exp OR 'nonbacterial thrombotic endocarditis'/exp OR coagulopath*:ti,ab,kw OR hypercoagulab*:ti,ab,kw OR thrombophili*:ti,ab,kw OR 'Trousseau syndrome':ti,ab,kw OR 'disseminated intravascular coagulation':ti,ab,kw OR 'nonbacterial thrombotic endocarditis':ti,ab,kw OR 'non-bacterial thrombotic endocarditis':ti,ab,kw OR 'marantic endocarditis':ti,ab,kw) ) OR 'cancer-associated stroke':ti,ab,kw OR 'cancer associated stroke':ti,ab,kw OR 'cancer-related stroke':ti,ab,kw OR 'cancer related stroke':ti,ab,kw OR 'cancer-associated ischemic stroke':ti,ab,kw OR 'cancer-associated ischaemic stroke':ti,ab,kw OR 'cancer-associated hypercoagulation':ti,ab,kw OR 'cancer-associated hypercoagulability':ti,ab,kw OR 'cancer-associated coagulopathy':ti,ab,kw OR ('Trousseau syndrome':ti,ab,kw AND (stroke:ti,ab,kw OR infarct*:ti,ab,kw)) |
| Fields | MeSH; Title/Abstract [tiab]. | TITLE-ABS-KEY. | Title, abstract, keywords (ti,ab,kw) plus MeSH descriptors. | Title, abstract, author keywords (:ti,ab,kw) plus exploded Emtree terms (/exp). |
| Dates | January 2000 to December 2025 | January 2000 to December 2025 | January 2000 to December 2025 | January 2000 to December 2025 |
| Language Limits | English OR Spanish | English OR Spanish | English OR Spanish | English OR Spanish |
