# Supplementary material for: Stroke secondary to cancer-associated coagulopathy: a systematic review
Source: J Neurol. 2026 Sep 20;273(10):609. doi: 10.1007/s00415-026-14119-y (PMC13590450; doi:10.1007/s00415-026-14119-y)
Supplement: Supplementary file 2 [file 415_2026_14119_MOESM2_ESM.docx]

**STROKE SECONDARY TO CANCER-ASSOCIATED COAGULOPATHY: A SYSTEMATIC REVIEW.**

Jauregui Larrañaga C^1,2#^, Gómez Arteta E^3^, Marta Enguita J^4,5*^, Erro Aguirre ME^6,7*^

^1^Neurology Department. Hospital Universitario Donostia. Donostia-San Sebastián.

^2^Biogipuzkoa Health Research Institute. Donostia-San Sebastián.

^3^Hematology Department. Hospital Universitario de Navarra. Pamplona.

^4^Neurology Department. Hospital Universitario Marqués de Valdecilla. Santander.

^5^Neurological Clinical Research Group, Instituto Investigación Sanitaria Valdecilla (IDIVAL), Santander.

^6^Neurology Department. Hospital Universitario de Navarra (HUN). Pamplona.

^7^Neuroepigenetics Laboratory-Navarrabiomed-Universidad Pública de Navarra (UPNA)-Instituto de investigación sanitaria de Navarra (IdiSNA), Pamplona.

*These authors share senior co-authorship.

Article type: Systematic review

(#) author of correspondence:

Carlota Jauregui Larrañaga, MD

Neurology Department.

Hospital Universitario Donostia

Begiristain Doktorea Pasealekua, 20014 Donostia (Gipuzkoa)

Tel.: +943 007000

e-mail: carlotajaureguilarranaga@gmail.com

https://orcid.org/**0000-0002-7963-5169**

**Supplementary Table 2.** Methodological quality and risk of bias assessment of included observational studies using the Newcastle-Ottawa Scale (NOS).

|  | **Author (year)** | **Study Type** | **Cancer**  **Definition** | **Stroke**  **Ascertainment** | **Cancer Types**  **(% AdenoCa)** | **Selection (Max 4★)** | **Comp. (Max 2★)** | **Outcome (Max 3★)** | **Quality** |
| --- | --- | --- | --- | --- | --- | --- | --- | --- | --- |
|  | **Taccone FS (2008)**^1^ | Retrospective  (Stroke cohort) | Pathologically confirmed | Clinical  + Imaging | **Multi-system.** *  (Unspecified) | ★★★ | ★ | ★ | **Low-Mod** |
|  | **Kim SG (2010)**^2^ | Prospective  (Stroke/Cancer) | Clinical Dx | MRI (DWI) | **Multi-system.**  (Unspecified) | ★★★★ | ★★ | ★★ | **High** |
|  | **Seok JM (2010)**^3^ | Prospective (Stroke/Cancer) | Pathologically confirmed | MRI (DWI) | **Multi-system.**  (Unspecified) | ★★★★ | ★★ | ★★ | **High** |
|  | **Zöller B (2012)**^4^ | Retrospective  (Cancer cohort) | Sweden Registry | ICD-9/10 codes  (isch. and hem.) | **Multi-system.** *  (Unspecified) | ★★★★ | ★★ | ★★★ | **High** |
|  | **Schwarzbach CJ (2012)**^5^ | Retrospective case-control  (Stroke/Cancer) | Medical records  AC if <6m | Clinical  + Imaging | **Multi-system.** *  (mainly Adenocarcinoma) | ★★★★ | ★★ | ★★ | **High** |
|  | **Seok JM (2012)**^6^ | Prospective (Stroke/Cancer) | Medical records  AC if <6m | MRI (DWI) | **Unspecified.**  (Unspecified) | ★★★★ | ★★ | ★★ | **High** |
|  | **Kono T (2012)**^7^ | Retrospective  (Stroke cohort) | Pathologically confirmed.  AC if <5y  Recent dx if <1y | MRI (DWI) | **Multi-system.** *  (Unspecified) | ★★★ | ★ | ★★ | **Moderate** |
|  | **Navi BB (2014)**^8^ | Retrospective (Stroke/Cancer) | Medical records  AC if <6m | ICD-9/10 codes | **Multi-system.** *  (60% AdenoCa) | ★★★ | ★★ | ★★ | **High** |
|  | **Navi BB (2014)**^9^ | Retrospective (Stroke/Cancer) | Active systemic Dx | ICD-9/10 codes | **Multi-system.** *  (60% AdenoCa) | ★★★ | ★★ | ★★ | **High** |
|  | **Guo YJ (2014)**^10^ | Retrospective  (Stroke cohort) | Medical records  AC if <1y | MRI (DWI) | **Multi-system.** *  (55% AdenoCa) | ★★★ | ★ | ★★ | **Moderate** |
|  | **Cocho D (2015)**^11^ | Retrospective (Stroke/Cancer) | Pathologically confirmed | Clinical  + Imaging | **Multi-system.** *  (Unspecified) | ★★★ | ★ | ★★ | **Moderate** |
|  | **Navi BB (2015)**^12^ | Retrospective (Cancer cohort) | Medical records | ICD-9/10 codes | **Breast, Colorectal, Lung, Pancreas, and prostate** (mainly Adenocarcinoma) | ★★★★ | ★★ | ★★★ | **High** |
|  | **Schwarzbach CJ (2015)**^13^ | Retrospective (Stroke/Cancer) | Medical records | MRI (DWI) | **Multi-system.**  (Unspecified) | ★★★ | ★ | ★★ | **Moderate** |
|  | **Karlińska AG (2015)**^14^ | Prospective  (Stroke cohort) | Medical records  AC if <1y | Clinical  + Imaging | **Multi-system.** *  (Unspecified) | ★★★★ | ★★ | ★★ | **High** |
|  | **Jang H (2015)**^15^ | Retrospective (Stroke/Cancer) | Medical records  AC if <6m | MRI (DWI) | **Multi-system.** *  (68% AdenoCa) | ★★★ | ★ | ★★ | **Moderate** |
|  | **Kneihsl M (2016)**^16^ | Retrospective  (Stroke cohort) | Medical records | Clinical  + Imaging | **Multi-system.**  (61% AdenoCa) | ★★★ | ★ | ★★ | **Moderate** |
|  | **Gon Y (2016)**^17^ | Retrospective  (Stroke cohort) | Medical records  AC if <6m | MRI (DWI) | **Multi-system.** *  (mainly Adenocarcinoma) | ★★★★ | ★★ | ★★ | **High** |
|  | **Thålin C (2016)**^18^ | Prospective case-control  (Stroke cohorts) | Medical records  AC if <6m | Clinical  + Imaging | **Breast, Colorectal, Lung, Pancreas, Urothelial and Prostate.**  (88% AdenoCa) | ★★★ | ★ | ★★ | **Moderate** |
|  | **Bang OY (2016)**^19^ | Prospective (Stroke/Cancer) | Pathologically confirmed | MRI (DWI) | **Multi-system.**  (Unespecified) | ★★★★ | ★★ | ★★ | **High** |
|  | **Finelli PF (2016)**^20^ | Retrospective  (restricted-DWI) | Medical records | MRI (DWI) | **Lung, colon, renal, pancreas, bladder.** (Unespecified) | ★★★ | ★ | ★ | **Moderate** |
|  | **Shin YW (2016)**^21^ | Retrospective (Stroke/Cancer) | Medical records  AC if <6m | MRI (DWI) | **Multi-system.**  (87% AdenoCa) | ★★★★ | ★ | ★★ | **High** |
|  | **Lee MJ (2017)**^22^ | Prospective (Stroke/Cancer) | Pathologically confirmed | MRI (DWI) | **Multi-system.**  (66% AdenoCa) | ★★★★ | ★★ | ★★★ | **High** |
|  | **Nam (2017)**^23^ | Retrospective (Stroke/Cancer) | Medical records  AC if <6m | MRI (DWI) | **Multi-system.**  (Unspecified) | ★★★★ | ★★ | ★★ | **High** |
|  | **Cutting S (2017)**^24^ | Retrospective  (Stroke cohort) | Medical records | Clinical  + Imaging | **Multi-system.**  (Unspecified) | ★★★ | ★ | ★★ | **Moderate** |
|  | **Selvik HA (2018)**^25^ | Prospective  (Stroke cohort) | Medical records  AC if <1y | MRI (DWI) | **Multi-system.**  (Unspecified) | ★★★★ | ★★ | ★★ | **High** |
|  | **Quintas S (2018)**^26^ | Retrospective  (Stroke cohort) | Pathologically confirmed | MRI (DWI) | **Multi-system.** *  (62% AdenoCa) | ★★★ | ★★ | ★★ | **High** |
|  | **Grazioli S (2018)**^27^ | Retrospective  (Stroke cohort) | Medical records  AC if <6m | Clinical  + Imaging | **Multi-system.** *  (Unspecified) | ★★★★ | ★★ | ★★ | **High** |
|  | **Chung (2018)**^28^ | Prospective (Stroke/Cancer) | Pathologically confirmed | Clinical  + Imaging | **Lung.**  (83% AdenoCa) | ★★★★ | ★★ | ★★ | **High** |
|  | **Wang J (2018)**^29^ | Retrospective case-control  (Stroke cohort) | Medical records  AC if <6m | MRI (DWI) | **Multi-system.** *  (Unspecified) | ★★★ | ★ | ★★ | **Moderate** |
|  | **Nam KW (2018)**^30^ | Retrospective  (Stroke/Cancer) | Medical records  AC if <6m | MRI (DWI) | **Multi-system.**  (67% AdenoCa) | ★★★★ | ★★ | ★★ | **High** |
|  | **Xiang E (2018)**^31^ | Retrospective  (Cancer cohort) | Medical records  AC if <1y | Clinical  + Imaging | **Multi-system.** *  (Unspecified) | ★★★ | ★ | ★★ | **Moderate** |
|  | **Park H (2019)**^32^ | Prospective (Stroke/Cancer) | Medical records  AC if <6m | Clinical  + Imaging. | **Multi-system.** *  (Unspecified) | ★★★★ | ★ | ★★ | **High** |
|  | **Bang OY (2019)**^33^ | Prospective (Stroke/Cancer) | Medical records  AC if <6m | MRI (DWI) | **Unspecified.**  (Unspecified) | ★★★ | ★ | ★★ | **Moderate** |
|  | **Nouh AM (2019)**^34^ | Retrospective  (Stroke cohort) | Medical records | MRI (DWI) | **Multi-system.**  (Unspecified) | ★★ | ★ | ★ | **Moderate** |
|  | **Ren R (2020)**^35^ | Retrospective (Stroke/Cancer) | Pathologically confirmed | MRI (DWI) | **Multi-system.**  (53% AdenoCa) | ★★★ | ★ | ★★ | **Moderate** |
|  | **Zhang J (2020)**^36^ | Retrospective case-control  (Stroke cohorts) | Medical records | MRI (DWI) | **Lung.**  (Unspecified) | ★★★ | ★ | ★★ | **Moderate** |
|  | **Yoo J (2020)**^37^ | Retrospective (Stroke/Cancer) | Medical records  AC if <6m | MRI (DWI) | **Multi-system.**  (Unspecified) | ★★★ | ★ | ★★ | **Moderate** |
|  | **Fu CH (2020)**^38^ | Retrospective  (Stroke cohort) | Medical records  AC if <6m | Clinical  + Imaging. | **Multi-system.**  (62% AdenoCa) | ★★★ | ★ | ★★ | **Moderate** |
|  | **Guo L (2020)**^39^ | Retrospective  (Stroke cohort) | Medical records  AC if <6m | MRI (DWI) | **Multi-system.**  (75% AdenoCa) | ★★★ | ★ | ★★ | **Moderate** |
|  | **Bao L (2020)**^40^ | Retrospective  (Stroke cohort) | Pathologically confirmed | MRI (DWI) | **Multi-system.**  (All AdenoCa) | ★★★★ | ★★ | ★★ | **High** |
|  | **Kawano T (2021)**^41^ | Retrospective  (Cancer cohort) | Medical records  AC if <2y | Medical records | **Multi-system.**  (Unspecified) | ★★★★ | ★★ | ★★ | **High** |
|  | **Maezono-Kandori K (2021)**^42^ | Retrospective  (Stroke cohort) | Medical records  AC if <6m | MRI (DWI) | **Multi-system.**  (Unspecified) | ★★★ | ★★ | ★★ | **High** |
|  | **Nakajima S (2022)**^43^ | Retrospective (Stroke/Cancer) | Medical records  AC if <6m | MRI (DWI) | **Multi-system.**  (62% AdenoCa) | ★★★★ | ★★ | ★★ | **High** |
|  | **Nezu T (2022)**^44^ | Prospective (Stroke/Cancer) | Medical records  AC if <6m | MRI (DWI) | **Multi-system.**  (Unspecified) | ★★★★ | ★★ | ★★ | **High** |
|  | **Navi BB (2022)**^45^ | Prospective (Stroke/Cancer) | Medical records  AC if <6m | MRI (DWI) | **Multi-system**  (88% AdenoCa) | ★★★★ | ★★ | ★★ | **High** |
|  | **Kataoka Y (2022)**^46^ | Retrospective  (Stroke cohort) | Medical records | Clinical  + Imaging. | **Multi-system**  (82% AdenoCa) | ★★★★ | ★★ | ★★ | **High** |
|  | **Finelli PF (2022)**^47^ | Prospective  (Stroke cohort) | Pathologically confirmed | MRI (DWI) | **Multi-system**  (56% AdenoCa) | ★★★ | ★ | ★★ | **Moderate** |
|  | **Tanimura J (2022)**^48^ | Retrospective (Stroke/Cancer) | Medical records  AC if <6m | MRI (DWI) | **Multi-system.**  (56% AdenoCa) | ★★★ | ★ | ★★ | **Moderate** |
|  | **Costamagna G (2023)**^49^ | Retrospective  (Stroke cohort) | Medical records  AC if <6m | Clinical  + Imaging. | **Multi-system.** *  (Unspecified) | ★★★★ | ★★ | ★★ | **High** |
|  | **Bravo-Anguiano Y (2023)**^50^ | Retrospective case-control  (Stroke cohort) | Medical records  AC if <1y | Clinical  + Imaging. | **Multi-system.** *  (Unspecified) | ★★★ | ★ | ★★ | **Moderate** |
|  | **Cen G (2023)**^51^ | Prospective (Stroke/Cancer) | Pathologically confirmed  AC if <6m | MRI (DWI) | **Liver**  (Hepatocellular) | ★★★★ | ★★ | ★★ | **High** |
|  | **Feldman S (2023)**^52^ | Retrospective  (Cancer cohort) | Medical records | Medical records | **Multi-system.**  (Unspecified) | ★★★ | ★★ | ★★ | **High** |
|  | **Beyeler M (2023)**^53^ | Retrospective  (Stroke cohort) | Medical records  AC if <1y | Clinical  + Imaging. | **Multi-system.** *  (58% AdenoCa) | ★★★★ | ★★ | ★★ | **High** |
|  | **Gon Y (2023)**^54^ | Prospective (Stroke/Cancer) | Medical records  AC if <6m | Clinical  + Imaging | **Multi-system**  (55% AdenoCa) | ★★★★ | ★★ | ★★ | **High** |
|  | **Ohno M (2023)**^55^ | Retrospective  (Stroke cohort) | Medical records  AC if <6m | MRI (DWI) | **Multi-system.** *  (Unspecified) | ★★★ | ★ | ★★ | **Moderate** |
|  | **Fujinami J (2023)**^56^ | Retrospective (Stroke/Cancer) | Medical records  AC if <6m | MRI (DWI) | **Multi-system**  (71% AdenoCa) | ★★★ | ★★ | ★★ | **High** |
|  | **Gon Y (2024)**^57^ | Retrospective  (Cancer cohort) | Medical records | ICD-10 codes | **Multi-system.** *  (Unspecified) | ★★★★ | ★★ | ★★ | **High** |
|  | **Acir I (2024)**^58^ | Retrospective (Stroke/Cancer) | Medical records | Clinical  + Imaging. | **Multi-system.**  (Unspecified) | ★★★ | ★ | ★★ | **Moderate** |
|  | **Patrzalek (2024)**^59^ | Retrospective  (Cancer cohort) | Medical records | Clinical  + Imaging | **Multi-system.** *  (76% AdenoCa) | ★★★ | ★★ | ★★ | **High** |
|  | **Cen G (2024)**^60^ | Prospective (Stroke/Cancer) | Pathologically confirmed  AC if <6m. | MRI (DWI) | **Multi-system**  (50% AdenoCa) | ★★★★ | ★★ | ★★ | **High** |
|  | **Fukunaga D (2024)**^61^ | Retrospective  (Stroke cohort) | Medical records  AC if <6m | MRI (DWI) | **Multi-system.**  (Unspecified) | ★★★ | ★ | ★★ | **Moderate** |
|  | **Navi BB (2024)**^62^ | Retrospective  (Stroke cohort) | Medical records | Clinical  + Imaging | **Multi-system.**  (Unspecified) | ★★★ | ★★ | ★★ | **High** |
|  | **Kawano T (2024)**^63^ | Prospective (Stroke/Cancer) | Medical records  AC if <6m | Clinical  + Imaging | **Multi-system.** *  (Unspecified) | ★★★★ | ★★ | ★★ | **High** |
|  | **Zhao B (2024)**^64^ | Retrospective (Stroke/Cancer) | Medical records | MRI (DWI) | **Multi-system.**  (Unspecified) | ★★★ | ★ | ★★ | **Moderate** |
|  | **Oh MS (2025)**^65^ | Prospective  (Stroke cohort) | Medical records | MRI (DWI) | **Multi-system.**  (Unspecified) | ★★★★ | ★★ | ★★★ | **High** |
|  | **Oka K (2025)**^66^ | Retrospective  (Cancer cohort) | Pathologically confirmed | Clinical  + Imaging | **Pancreas, Gastric.** (AdenoCa) | ★★★ | ★★ | ★★ | **High** |
|  | **Otani T (2025)**^67^ | Retrospective (NBTE cohort) | Medical records | Pathologically confirmed | **Multi-system.** *  (81% AdenoCa) | ★★★ | ★ | ★★ | **Moderate** |
|  | **Hyun JW (2025)**^68^ | Retrospective  (Cancer cohort) | Medical records | MRI (DWI) | **Multi-system.**  (Unspecified)* | ★★★★ | ★★ | ★★ | **High** |
|  | **Woock M (2025)**^69^ | Retrospective case-control  (Stroke cohort) | Medical records  AC if <6m | Clinical  + Imaging. | **Multi-system.** *  (Unspecified) | ★★★★ | ★★ | ★★ | **High** |
|  | **Kim TJ (2025)**^70^ | Prospective  (Stroke cohort) | Medical records | Clinical  + Imaging | **Unspecified.**  (Unspecified) | ★★★ | ★ | ★★ | **Moderate** |
|  | **Oakley CI (2025)**^71^ | Retrospective  (Cancer case series) | Medical records | MRI (DWI) | **Multi-system** *  (74% AdenoCa) | ★★★ | ★★ | ★★ | **High** |
|  | **Fu CH (2025)**^72^ | Prospective  (Stroke cohort) | Medical records  AC if <6m | Clinical  + Imaging. | **Multi-system**  (62% AdenoCa) | ★★★ | ★ | ★★ | **Moderate** |
|  | **Chandu M (2025)**^73^ | Retrospective  (Stroke cohort) | Medical records | Clinical  + Imaging | **Multi-system.** *  (Unspecified) | ★★★ | ★ | ★★ | **Moderate** |
|  | **Kim TJ (2025)**^74^ | Retrospective (Stroke/Cancer) | Medical records | Clinical  + Imaging. | **Multi-system.** *  (Unspecified) | ★★★ | ★ | ★★ | **Moderate** |
|  | **Kielkopf MC (2025)**^75^ | Retrospective (Stroke/Cancer) | Medical records  AC if <6m | Clinical  + Imaging | **Multi-system.** *  (Unspecified) | ★★★★ | ★ | ★★ | **High** |
|  | **Xie L (2025)**^76^ | Retrospective case-control  (Stroke cohort) | Medical records | Clinical  + Imaging. | **Multi-system.** *  (61% AdenoCa) | ★★★★ | ★★ | ★★ | **High** |
|  | **Ke X (2025)**^77^ | Retrospective  (Cancer cohort) | Medical records | MRI (DWI) | **Multi-system.**  (Unspecified) | ★★★ | ★ | ★★ | **Moderate** |
|  | **Gon (2026)**^78^ | Prospective (Stroke/Cancer) | Medical records  AC if <6m | MRI (DWI) | **Multi-system.**  (55% AdenoCa) | ★★★★ | ★★ | ★★ | **High** |

*Comp.: Comparative; MRI: magnetic resonance image; DWI: diffuse-weighted image; AC: active cancer.*

**Include hematologic malignancies, ranging from 0% to 20% across studies.*

**Supplementary Table 3.** Methodological quality and risk of bias assessment of included randomized clinical trials using the Risk Of Bias version 2 (RoB-2) tool.

| **Reference** | **Study Design** | **Comparison & Population** | **D1 / Confounding** | **D2 /**  **Interventions** | **D3 /**  **Missing Data** | **D4 /**  **Measurement** | **D5 /**  **Reporting** |
| --- | --- | --- | --- | --- | --- | --- | --- |
| **Navi BB et al. (TEACH, 2018)**^79^ | Open-Label RCT | Enoxaparin (n=10) vs. Aspirin (n=10) in active cancer with AIS | **Low Risk** | **High Risk**  *(Open-label design)* | **Low Risk** | **Low Risk**  *(Blinded assessors)* | **Low Risk** |
| **Chung JW et al. (ENCHASE, 2024)**^80^ | Open-Label RCT | Edoxaban (n=20) vs. Enoxaparin (n=20) in cancer-related ESUS | **Low Risk** | **High Risk**  *(Open-label design)* | **Some Concerns** *(COVID-19 missed visits)* | **Low Risk**  *(Blinded outcome consensus)* | **Low Risk** |

*RCT: randomized clinical trial; AIS: acute ischemic stroke; ESUS: embolic stroke of undetermined source.*

**Supplementary Table 4.** Methodological quality and risk of bias assessment of included experimental animal studies using the Systematic Review Centre for Laboratory animal Experimentation (SYRCLE) tool.

| **Reference** | **Experimental Animal Model** | **Tumor / Cell Lines Used** | **Selection Bias (Sequence & Baseline)** | **Allocation Concealment** | **Performance Bias (Housing & Blinding)** | **Detection Bias (Outcome Assessment)** | **Attrition & Reporting** | **Overall Risk of Bias** |
| --- | --- | --- | --- | --- | --- | --- | --- | --- |
| **Shao et al (2011)**^81^ | C57BL/6J (PSGL-1–/–, PAR4–/–, P-sel–/–, CG–/–, NE–/–, Hck–/–/Fgr–/–/Lyn–/–) | Carcinoma mucins (disseminated Trousseau syndrome model) | **Moderate** *(Littermate controls backcrossed 10 generations)* | **Unspecified** | **Unspecified** | **Unspecified** | **Low Risk** | **Moderate-High** |
| **Chou et al. (2025)**^82^ | F8–/– (HA), F9–/– (HB), VWD (VWF R1326H), NSG, NSG-HA mice | Orthotopically injected pancreatic cancer lines (AsPC-1, MIA PaCa-2) | **Moderate** *(Wild-type littermates used as controls)* | **Unspecified** | **Unspecified** | **Unspecified** | **Low Risk** | **Moderate-High** |

**REFERENCES**

1.	Taccone FS, Jeangette SM, Blecic SA. First-Ever Stroke as Initial Presentation of Systemic Cancer. *J Stroke Cerebrovasc Dis*. 2008;17(4):169-174. doi:10.1016/j.jstrokecerebrovasdis.2008.01.007

2.	Kim SG, Hong JM, Kim HY, et al. Ischemic stroke in cancer patients with and without conventional mechanisms: a multicenter study in Korea. *Stroke*. 2010;41(4):798-801. doi:10.1161/STROKEAHA.109.571356

3.	Seok JM, Kim SG, Kim JW, et al. Coagulopathy and embolic signal in cancer patients with ischemic stroke. *Ann Neurol*. 2010;68(2):213-219. doi:10.1002/ana.22050

4.	Zöller B, Ji J, Sundquist J, Sundquist K. Risk of haemorrhagic and ischaemic stroke in patients with cancer: a nationwide follow-up study from Sweden. *Eur J Cancer*. 2012;48(12):1875-1883. doi:10.1016/j.ejca.2012.01.005

5.	Schwarzbach CJ, Schaefer A, Ebert A, et al. Stroke and cancer: the importance of cancer-associated hypercoagulation as a possible stroke etiology. *Stroke*. 2012;43(11):3029-3034. doi:10.1161/STROKEAHA.112.658625

6.	Seok JM, Kim SJ, Song P, et al. Clinical presentation and ischemic zone on MRI in cancer patients with acute ischemic stroke. *Eur Neurol*. 2012;68(6):368-376. doi:10.1159/000341147

7.	Kono T, Ohtsuki T, Hosomi N, et al. Cancer-associated ischemic stroke is associated with elevated D-dimer and fibrin degradation product levels in acute ischemic stroke with advanced cancer. *Geriatr Gerontol Int*. 2012;12(3):468-474. doi:10.1111/j.1447-0594.2011.00796.x

8.	Navi BB, Singer S, Merkler AE, et al. Cryptogenic subtype predicts reduced survival among cancer patients with ischemic stroke. *Stroke*. 2014;45(8):2292-2297. doi:10.1161/STROKEAHA.114.005784

9.	Navi BB, Singer S, Merkler AE, et al. Recurrent thromboembolic events after ischemic stroke in patients with cancer. *Neurology*. 2014;83(1):26-33. doi:10.1212/WNL.0000000000000539

10.	Guo YJ, Chang MH, Chen PL, Lee YS, Chang YC, Liao YC. Predictive value of plasma (D)-dimer levels for cancer-related stroke: a 3-year retrospective study. *J stroke Cerebrovasc Dis Off J Natl Stroke Assoc*. 2014;23(4):e249-54. doi:10.1016/j.jstrokecerebrovasdis.2013.10.022

11.	Cocho D, Gendre J, Boltes A, et al. Predictors of occult cancer in acute ischemic stroke patients. *J stroke Cerebrovasc Dis Off J Natl Stroke Assoc*. 2015;24(6):1324-1328. doi:10.1016/j.jstrokecerebrovasdis.2015.02.006

12.	Navi BB, Reiner AS, Kamel H, et al. Association between incident cancer and subsequent stroke. *Ann Neurol*. 2015;77(2):291-300. doi:10.1002/ana.24325

13.	Schwarzbach CJ, Fatar M, Eisele P, Ebert AD, Hennerici MG, Szabo K. DWI lesion patterns in cancer-related stroke – specifying the phenotype. *Cerebrovasc Dis Extra*. 2015;5(3):139-145. doi:10.1159/000439549

14.	Karlińska AG, Gromadzka G, Karliński MA, Członkowska A. The activity of malignancy may determine stroke pattern in cancer patients. *J stroke Cerebrovasc Dis Off J Natl Stroke Assoc*. 2015;24(4):778-783. doi:10.1016/j.jstrokecerebrovasdis.2014.11.003

15.	Jang H, Lee JJ, Lee MJ, et al. Comparison of Enoxaparin and Warfarin for Secondary Prevention of Cancer-Associated Stroke. *J Oncol*. 2015;2015(July 2006). doi:10.1155/2015/502089

16.	Kneihsl M, Enzinger C, Wünsch G, et al. Poor short-term outcome in patients with ischaemic stroke and active cancer. *J Neurol*. 2016;263(1):150-156. doi:10.1007/s00415-015-7954-6

17.	Gon Y, Okazaki S, Terasaki Y, et al. Characteristics of cryptogenic stroke in cancer patients. *Ann Clin Transl Neurol*. 2016;3(4):280-287. doi:10.1002/acn3.291

18.	Thålin C, Demers M, Blomgren B, et al. NETosis promotes cancer-associated arterial microthrombosis presenting as ischemic stroke with troponin elevation. *Thromb Res*. 2016;139:56-64. doi:10.1016/j.thromres.2016.01.009

19.	Bang OY, Chung JW, Lee MJ, et al. Cancer cell-derived extracellular vesicles are associated with coagulopathy causing ischemic stroke via tissue factor-independent way: The OASIS-CANCER study. *PLoS One*. 2016;11(7):1-15. doi:10.1371/journal.pone.0159170

20.	Finelli PF, Nouh A. Three-Territory DWI acute infarcts: Diagnostic value in cancer-Associated hypercoagulation stroke (trousseau syndrome). *Am J Neuroradiol*. 2016;37(11):2033-2036. doi:10.3174/ajnr.A4846

21.	Shin YW, Lee ST, Jung KH, et al. Predictors of survival for patients with cancer after cryptogenic stroke. *J Neurooncol*. 2016;128(2):277-284. doi:10.1007/s11060-016-2106-0

22.	Lee MJ, Chung JW, Ahn MJ, et al. Hypercoagulability and Mortality of Patients with Stroke and Active Cancer: The OASIS-CANCER Study. *J stroke*. 2017;19(1):77-87. doi:10.5853/jos.2016.00570

23.	Nam KW, Kim CK, Kim TJ, et al. D-dimer as a predictor of early neurologic deterioration in cryptogenic stroke with active cancer. *Eur J Neurol*. 2017;24(1):205-211. doi:10.1111/ene.13184

24.	Cutting S, Wettengel M, Conners JJ, Ouyang B, Busl K. Three-Month Outcomes Are Poor in Stroke Patients with Cancer Despite Acute Stroke Treatment. *J Stroke Cerebrovasc Dis*. 2017;26(4):809-815. doi:10.1016/j.jstrokecerebrovasdis.2016.10.021

25.	Selvik HA, Bjerkreim AT, Thomassen L, Waje-Andreassen U, Naess H, Kvistad CE. When to Screen Ischaemic Stroke Patients for Cancer. *Cerebrovasc Dis*. 2018;45(1-2):42-47. doi:10.1159/000484668

26.	Quintas S, Rogado J, Gullón P, et al. Predictors of unknown cancer in patients with ischemic stroke. *J Neurooncol*. 2018;137(3):551-557. doi:10.1007/s11060-017-2741-0

27.	Grazioli S, Paciaroni M, Agnelli G, et al. Cancer-associated ischemic stroke: A retrospective multicentre cohort study. *Thromb Res*. 2018;165:33-37. doi:10.1016/j.thromres.2018.03.011

28.	Chung JW, Cho YH, Ahn MJ, et al. Association of cancer cell type and extracellular vesicles with coagulopathy in patients with lung cancer and stroke. *Stroke*. 2018;49(5):1282-1285. doi:10.1161/STROKEAHA.118.020995

29.	Wang J ye, Zhang G jia, Zhuo S xia, et al. D-dimer >2.785 μg/ml and multiple infarcts ≥3 vascular territories are two characteristics of identifying cancer-associated ischemic stroke patients. *Neurol Res*. 2018;40(11):948-954. doi:10.1080/01616412.2018.1504179

30.	Nam KW, Kim TJ, Kim CK, et al. Temporal changes in the neutrophil to lymphocyte ratio and the neurological progression in cryptogenic stroke with active cancer. *PLoS One*. 2018;13(3):e0194286. doi:10.1371/journal.pone.0194286

31.	Xiang E, Ahuja T, Raco V, Cirrone F, Green D, Papadopoulos J. Anticoagulation prescribing patterns in patients with cancer. *J Thromb Thrombolysis*. 2018;45(1):89-98. doi:10.1007/s11239-017-1558-1

32.	Park H, Kim J, Ha J, et al. Histological features of intracranial thrombi in stroke patients with cancer. *Ann Neurol*. 2019;86(1):143-149. doi:10.1002/ana.25495

33.	Bang OY, Chung JW, Cho YH, et al. Circulating DNAs, a Marker of Neutrophil Extracellular Traposis and Cancer-Related Stroke: The OASIS-Cancer Study. *Stroke*. 2019;50(10):2944-2947. doi:10.1161/STROKEAHA.119.026373

34.	Nouh AM, Staff I, Finelli PF. Three Territory Sign: An MRI marker of malignancy-related ischemic stroke (Trousseau syndrome). *Neurol Clin Pract*. 2019;9(2):124-128. doi:10.1212/CPJ.0000000000000603

35.	Ren R, Yan H, Gui Y, et al. Clinical Features of Trousseau Syndrome With Cerebral Infarction as the Initial Manifestation. *Neurologist*. 2020;25(5):117-121. doi:10.1097/NRL.0000000000000275

36.	Zhang J, Zhao J. Clinical Characteristics and Analysis of Lung Cancer- Associated Acute Ischemic Stroke. *J stroke Cerebrovasc Dis Off J Natl Stroke Assoc*. 2020;29(10):105164. doi:10.1016/j.jstrokecerebrovasdis.2020.105164

37.	Yoo J, Choi JK, Kim YD, et al. Outcome of Stroke Patients with Cancer and Nonbacterial Thrombotic Endocarditis. *J Stroke*. 2020;22(2):245-253. doi:10.5853/jos.2020.00619

38.	Fu CH, Chen CH, Lin YH, et al. Fibrin and Platelet-Rich Composition in Retrieved Thrombi Hallmarks Stroke With Active Cancer. *Stroke*. 2020;51(12):3723-3727. doi:10.1161/STROKEAHA.120.032069

39.	Guo L, Wang L, Liu W. Ability of the number of territories involved on DWI-MRI to predict occult systemic malignancy in cryptogenic stroke patients. *J Stroke Cerebrovasc Dis*. 2020;29(7):104823. doi:10.1016/j.jstrokecerebrovasdis.2020.104823

40.	Bao L, Zhang S, Gong X, Cui G. Trousseau Syndrome Related Cerebral Infarction: Clinical Manifestations, Laboratory Findings and Radiological Features. *J stroke Cerebrovasc Dis Off J Natl Stroke Assoc*. 2020;29(9):104891. doi:10.1016/j.jstrokecerebrovasdis.2020.104891

41.	Kawano T, Sasaki T, Gon Y, et al. High neutrophil/lymphocyte ratio at cancer diagnosis predicts incidence of stroke in cancer patients. *Brain Commun*. 2021;3(2):fcab071. doi:10.1093/braincomms/fcab071

42.	Maezono-Kandori K, Ohara T, Fujinami J, Makita N, Tanaka E, Mizuno T. Elevated CA125 is Related to Stroke Due to Cancer- Associated Hypercoagulation. *J Stroke Cerebrovasc Dis*. 2021;30(12):106126. doi:10.1016/j.jstrokecerebrovasdis.2021.106126

43.	Nakajima S, Kawano H, Yamashiro K, et al. Post-Treatment Plasma D-Dimer Levels Are Associated With Short-Term Outcomes in Patients With Cancer-Associated Stroke. *Front Neurol*. 2022;13(April):1-9. doi:10.3389/fneur.2022.868137

44.	Nezu T, Hosomi N, Naito H, et al. Clinical characteristics and tumor markers in ischemic stroke patients with active cancer. *Intern Emerg Med*. 2022;17(3):735-741. doi:10.1007/s11739-021-02862-1

45.	Navi BB, Zhang C, Sherman CP, et al. Ischemic stroke with cancer: Hematologic and embolic biomarkers and clinical outcomes. *J Thromb Haemost*. 2022;20(9):2046-2057. doi:10.1111/jth.15779

46.	Kataoka Y, Sonoda K, Takahashi JC, et al. Histopathological analysis of retrieved thrombi from patients with acute ischemic stroke with malignant tumors. *J Neurointerv Surg*. 2022;14(5). doi:10.1136/neurintsurg-2020-017195

47.	Finelli PF. MR Three-Territory Sign in Cancer-Associated Hypercoagulation Stroke (Trousseau Syndrome): An Overlooked Diagnostic Feature: MR Three-Territory Sign. *Neurologist*. 2022;27(2):37-40. doi:10.1097/NRL.0000000000000356

48.	Tanimura J, Yamamoto T, Hashimoto T. Clinical features and prognostic factors in patients with cancer-associated multiple ischemic stroke: A retrospective observational study. *J stroke Cerebrovasc Dis Off J Natl Stroke Assoc*. 2022;31(12):106813. doi:10.1016/j.jstrokecerebrovasdis.2022.106813

49.	Costamagna G, Hottinger A, Milionis H, et al. Clinical and Demographic Characteristics, Mechanisms, and Outcomes in Patients With Acute Ischemic Stroke and Newly Diagnosed or Known Active Cancer. *Neurology*. 2023;100(24):E2477-E2489. doi:10.1212/WNL.0000000000207341

50.	Bravo-anguiano Y, Echavarría-iñiguez A, Madrigal-lkhou E, Muñoz-martín A. Ictus asociado a cáncer : estudio de prevalencia y factores predictores entre pacientes con ictus isquémico. 2023;76(6):189-195. doi:10.33588/rn.7606.2022414.

51.	Cen G, Song Y, Chen S, et al. The investigation on the hypercoagulability of hepatocellular carcinoma-related cerebral infarction with thromboelastography. *Brain Behav*. 2023;13(4):e2961. doi:10.1002/brb3.2961

52.	Feldman S, Gupta D, Navi BB, et al. Tumor Genomic Profile Is Associated With Arterial Thromboembolism Risk in Patients With Solid Cancer. *JACC CardioOncology*. 2023;5(2):246-255. doi:10.1016/j.jaccao.2023.01.009

53.	Beyeler M, Grunder L, Göcmen J, et al. Absence of susceptibility vessel sign and hyperdense vessel sign in patients with cancer-related stroke. *Front Neurol*. 2023;14(March):1-8. doi:10.3389/fneur.2023.1148152

54.	Gon Y, Sakaguchi M, Yamagami H, et al. Predictors of Survival in Patients With Ischemic Stroke and Active Cancer: A Prospective, Multicenter, Observational Study. *J Am Heart Assoc*. 2023;12(15):e029618. doi:10.1161/JAHA.123.029618

55.	Ohno M, Miyakita Y, Takahashi M, Yanagisawa S, Ozeki Y, Yonezawa H, Omura T, Satomi N, Mukai T, Kawaguchi Y NY. Assessment of clinical and functional outcome in patients with cancer and acute ischemic stroke. *Res Sq*. Published online 2023:1-15.

56.	Fujinami J, Nagakane Y, Fujikawa K, et al. D-dimer trends predict recurrent stroke in patients with cancer-related hypercoagulability. *Cerebrovasc Dis Extra*. Published online 2023. doi:10.1159/000535644

57.	Gon Y, Morishima T, Kawano T, et al. Arterial Thromboembolism in Japanese Patients With Cancer: Incidence, Predictors, and Survival Impact. *JACC CardioOncology*. 2024;6(2):283-297. doi:10.1016/j.jaccao.2024.01.006

58.	Acır İ, Erdoğan HA, Toka E, Yayla V. The Influence of Cancer Type on Stroke Incidence: A Retrospective Study. *Clin Med Insights Oncol*. 2024;18:11795549241308090. doi:10.1177/11795549241308090

59.	Patrzalek P, Wysokinski WE, Kurmann RD, et al. Cancer-associated non-bacterial thrombotic endocarditis-Clinical series from a single institution. *Am J Hematol*. 2024;99(4):596-605. doi:10.1002/ajh.27239

60.	Cen G, Wang J, Wang X, et al. Pathogenesis and Biomarkers of Cancer-Related Ischemic Stroke. *J Inflamm Res*. 2024;17:8589-8597. doi:10.2147/JIR.S493406

61.	Fukunaga D, Fujinami J, Kishitani T, Tokuda N, Numa S, Nagakane Y. Absence of the Susceptibility Vessel Sign with Cancer-Associated Hypercoagulability-Related Stroke. *AJNR Am J Neuroradiol*. 2024;45(10):1427-1431. doi:10.3174/ajnr.A8363

62.	Navi BB, Zhang C, Miller B, et al. Apixaban vs Aspirin in Patients With Cancer and Cryptogenic Stroke: A Post Hoc Analysis of the ARCADIA Randomized Clinical Trial. *JAMA Neurol*. 2024;81(9):958-965. doi:10.1001/jamaneurol.2024.2404

63.	Kawano T, Gon Y, Sakaguchi M, et al. Von Willebrand Factor Antigen Levels Predict Poor Outcomes in Patients With Stroke and Cancer: Findings From the Multicenter, Prospective, Observational SCAN Study. *J Am Heart Assoc*. 2024;13(3):e032284. doi:10.1161/JAHA.123.032284

64.	Zhao B, Jia W, Yuan Y, Li Z. Clinical analysis of twenty ‑ one cases of acute ischemic stroke related to Trousseau syndrome. *Neurol Sci*. 2024;45(4):1537-1547. doi:10.1007/s10072-023-07180-2

65.	Oh MS, Yu KH, Lee BC, et al. Temporal patterns of occult cancer incidence following acute ischemic stroke: A comparative study by stroke etiology. *Int J stroke Off J Int Stroke Soc*. 2025;20(8):1031-1041. doi:10.1177/17474930251335615

66.	Oka K, Iwai N, Ohara T, et al. Ischemic stroke in patients with unresectable pancreatic and gastric cancer. *Int J Clin Oncol*. 2025;30(5):974-983. doi:10.1007/s10147-025-02731-3

67.	Otani T, Terada-Ikeda C, Koge J, et al. Vegetations and cerebral emboli of non-bacterial thrombotic endocarditis are overwhelmingly platelet-dominant: A possible histological clue for the diagnosis from an autopsy study. *Cardiovasc Pathol*. 2025;78:107746. doi:https://doi.org/10.1016/j.carpath.2025.107746

68.	Hyun JW, Payumo RMP, Chung J, et al. Neutrophil extracellular traposis in cancer patients with acute ischemic stroke. *PLoS One*. 2025;20(6):e0326654. doi:10.1371/journal.pone.0326654

69.	Woock M, Rossi R, Jabrah D, et al. Clot signature in patients with large vessel occlusion stroke and concomitant active cancer. *Eur J Neurol*. 2025;32(1):e70037. doi:10.1111/ene.70037

70.	Kim TJ, Jung JW, Kim YJ, Yoon BW, Han D, Ko SB. Proteomic Analyses of Clots Identify Stroke Etiologies in Patients Undergoing Endovascular Therapy. *CNS Neurosci Ther*. 2025;31(3):e70340. doi:10.1111/cns.70340

71.	Oakley CI, Wysokinska EM, Kaminska A, et al. Topography of ischemic strokes in cancer-associated non-bacterial thrombotic endocarditis: A single-institution descriptive case series. *J stroke Cerebrovasc Dis Off J Natl Stroke Assoc*. 2025;34(2):108171. doi:10.1016/j.jstrokecerebrovasdis.2024.108171

72.	Fu CH, Chen CH, Lin YH, et al. High fibrin and platelet clot predicts stroke recurrence or mortality after thrombectomy in patients with active cancer. *J Neurointerv Surg*. 2025;17(11):1189-1194. doi:10.1136/jnis-2024-022033

73.	Chandu M, Agarwal A, Vishnu VY, et al. Patterns and predictors of cancer-associated ischemic stroke. *J Neurosci Rural Pract*. 2025;16. doi:10.25259/JNRP_88_2025

74.	Kim TJ, Lee JS, Oh MS, et al. Long-Term Outcomes and Predictors in Cancer-Related Stroke Using the Linked Data Set. *J Am Heart Assoc*. 2025;14(16):e040883. doi:10.1161/JAHA.124.040883

75.	Kielkopf MC, Göcmen J, Venzin SB, et al. Anticoagulant versus antiplatelet treatment for secondary stroke prevention in patients with active cancer. *Front Neurol*. 2025;Volume 16-2025. doi:10.3389/fneur.2025.1530775

76.	Xie L, Hao K, Shen J, Huang C. Association of red blood cells, D-dimer, and 13-(S)-HODE with cancer-related ischemic stroke: a case-control study. *Am J Cancer Res*. 2025;15(9):4054-4066. doi:10.62347/RHLH9776

77.	Ke X, Wu X, Feng G, et al. Analysis of Prognostic Factors for Survival in Patients with Trousseau Syndrome Initially Presenting with Acute Ischemic Stroke. *Cerebrovasc Dis Extra*. 2025;15(1):202-209. doi:10.1159/000548109

78.	Gon Y, Kawano T, Kitano T, et al. Tissue factor activity and 1-year mortality in patients with active cancer and acute ischemic stroke: findings from the SCAN study. *Res Pract Thromb Haemost*. 2026;10(1):103347. doi:10.1016/j.rpth.2026.103347

79.	Navi BB, Marshall RS, Bobrow D, et al. Enoxaparin vs Aspirin in Patients With Cancer and Ischemic Stroke: The TEACH Pilot Randomized Clinical Trial. *JAMA Neurol*. 2018;75(3):379-381. doi:10.1001/jamaneurol.2017.4211

80.	Chung JW, Hwang J, Kim HJ, et al. Edoxaban for the treatment of hypercoagulability and cerebral thromboembolism associated with cancer: A randomized clinical trial of biomarker targets. *Int J Stroke*. 2024;19(6):645-653. doi:10.1177/17474930241239266

81.	Shao B, Wahrenbrock MG, Yao L, et al. Carcinoma mucins trigger reciprocal activation of platelets and neutrophils in a murine model of Trousseau syndrome. *Blood*. 2011;118(15):4015-4023. doi:10.1182/blood-2011-07-368514

82.	Chou SC, Chang SL, Yu CY, et al. Tissue factor, factor VIII and IX in microvesicle-induced thrombosis and tumor growth of pancreatic cancer. *Thromb J*. 2025;23(1):32. doi:10.1186/s12959-025-00715-x
