# Supplementary material for: Stroke secondary to cancer-associated coagulopathy: a systematic review
Source: J Neurol. 2026 Sep 20;273(10):609. doi: 10.1007/s00415-026-14119-y (PMC13590450; doi:10.1007/s00415-026-14119-y)
Supplement: Supplementary file 3 [file 415_2026_14119_MOESM3_ESM.docx]

**STROKE SECONDARY TO CANCER-ASSOCIATED COAGULOPATHY: A SYSTEMATIC REVIEW.**

Jauregui Larrañaga C^1,2#^, Gómez Arteta E^3^, Marta Enguita J^4,5*^, Erro Aguirre ME^6,7*^

^1^Neurology Department. Hospital Universitario Donostia. Donostia-San Sebastián.

^2^Biogipuzkoa Health Research Institute. Donostia-San Sebastián.

^3^Hematology Department. Hospital Universitario de Navarra. Pamplona.

^4^Neurology Department. Hospital Universitario Marqués de Valdecilla. Santander.

^5^Neurological Clinical Research Group, Instituto Investigación Sanitaria Valdecilla (IDIVAL), Santander.

^6^Neurology Department. Hospital Universitario de Navarra (HUN). Pamplona.

^7^Neuroepigenetics Laboratory-Navarrabiomed-Universidad Pública de Navarra (UPNA)-Instituto de investigación sanitaria de Navarra (IdiSNA), Pamplona.

*These authors share senior co-authorship.

Article type: Systematic review

(#) author of correspondence:

Carlota Jauregui Larrañaga, MD

Neurology Department.

Hospital Universitario Donostia

Begiristain Doktorea Pasealekua, 20014 Donostia (Gipuzkoa)

Tel.: +943 007000

e-mail: carlotajaureguilarranaga@gmail.com

https://orcid.org/**0000-0002-7963-5169**

**Supplementary Table 4.** Summary of review.

| **Cancer associated coagulopathy and stroke** | **Key findings/Results/Conclusions** | **References** |
| --- | --- | --- |
| Epidemiology | - Stroke can be the first manifestation of cancer in 5% of ischemic stroke patients - ATE among patients with cancer has a peak incidence one year after cancer diagnosis and stroke accounts for the majority (71.6%) - Occult malignancy is found in 5.3% of patients with stroke of undetermined cause and is notably higher in the early period following stroke - The risk of ischemic stroke in patients with cancer is increased during the first 3-6 months after diagnosis - Latency between cancer diagnosis and stroke is shorter in pancreatic and colorectal cancer - Adenocarcinoma is the most common cancer histology associated with stroke and lung, pancreatic and colorectal cancers are the most frequent cancers | Selvik HA 2018^1^  Gon Y 2024^2^  Cocho D 2015^3^, Oh MS 2025^4^  Zöller B 2012^5^, Navi BB 2015^6^, Quintas S 2018^7^  Acir I 2024^8^  Schwarzbach CJ 2012^9^, Navi BB 2014^10,11^, Navi BB 2015^6^, Kneihsl M 2016^12^, Nakajima S 2022^13^, Nezu T 2022^14^, Costamagna G 2023^15^, Oh MS 2025^4^, Gon Y 2016^16^, Bravo-Anguiano Y 2023^17^, Patrzalek 2024^18^, Zhao B 2024^19^, Oka K 2025^20^ |
| Clinical features | - Cancer patients with acute stroke caused by hypercoagulability present frequently with encephalopathy - Patients with cryptogenic stroke and active cancer had lower body mass index, fewer atherosclerotic risk factors, and lower nutritional status - Younger age, female sex, and metastatic cancer are associated with hypercoagulability comparing patients with acute stroke with newly diagnosed cancers vs those with known active cancer - Metastatic disease is significantly more frequent in patients with cancer with cryptogenic stroke and when stroke reveals a previously undiagnosed cancer - Patients with cancer and stroke face a very high risk of stroke recurrence | Seok JM 2012^21^  Gon Y 2016^16^; Tanimura J 2022^22^  Costamagna G 2023^15^  Taccone FS 2008^23^, Schwarzbach CJ 2012^9^, Navi BB 2014^10,11^, Gon Y 2016^16^, Quintas S 2018^7^, Ren R 2020^24^, Nezu T 2022^14^, Tanimura J 2022^22^  Navi BB 2014^11^, Nakajima S 2022^13^, Schwarzbach CJ 2015^25^, Costamagna G 2023^15^, Chandu M 2025^26^ |
| Pathogenesis | - Hypercoagulability (NBTE or disseminated intravascular coagulation) is the most frequent cancer related stroke mechanism - Cancer-related stroke hypercoagulability is mediated by cancer inflammation, endothelial and platelet dysfunction, and circulating microemboli - Thrombi in stroke patients with active cancer are platelet-rich and erythrocyte-poor, more evident in patients with NBTE - Alterations in K-ras and STK11 were associated with an increased risk of ATE - Pancreatic cancer thrombosis induced by TF-expressing microvesicles strongly depends on FVIII and FIX - Cancer-related stroke patients showed higher levels of NET-specific biomarkers such as plasma H3Cit levels and DNA levels - Cancer cell-derived EVs mediate coagulopathy resulting in ischemic stroke via TF-independent mechanisms - Shorter clotting times are dose-dependent after treatment with EVs from adenocarcinoma cell lines than with EVs from squamous cell carcinoma - vWF has the key role in the formation of clots in patients with active cancer and stroke - IGHG1 and VTN proteins associated with active cancer and immune responses are identified by proteomic analysis of clot composition of cancer related stroke patients | Taccone FS 2008^23^, Costamagna G 2023^15^, Zhang J 2020^27^, Cen G 2023^28^, Oka K 2025^20^, Navi BB 2014^10^, Yoo J 2020^29^, Grazioli S 2018^30^  Karlinska 2015^31^, Navi BB 2022^32^, Cen G 2024^33^  Park H 2019^34^, Kataoka Y 2022^35^, Fu CH 2020^36^, Otani T 2025^37^  Feldman S 2023^38^  Chou SC 2025^39^  Thålin C 2016^40^, Bang OY 2019^41^, Hyun JW 2025^42^  Bang OY 2016^43^  Chung JW 2018^44^  Woock M 2025^45^  Kim TJ 2025^46^ |
| Radiologic biomarkers | - Cryptogenic stroke in patients with cancer is associated with diffusion-weighted imaging lesion pattern of multiple vascular territories (“Three-territory sign”) - NBTE is most commonly associated with multifocal bihemispheric ischemic strokes of varying sizes involving both the anterior and posterior circulation - The absence of susceptibility vessel sign on MRI could help to identify paraneoplastic hypercoagulability in stroke patients with active cancer | Kim SG 2010^47^, Schwarzbach CJ 2012^9^, Schwarzbach CJ 2015^25^, Gon Y 2016^16^, Guo L 2020^48^, Finelli PF 2016^49^, Finelli PF 2022^50^, Guo YJ 2014^51^, Wang J 2018^52^, Nouh AM 2019^53^, Nezu T 2022^14^,Tanimura 2022 J^22^, Zhao B 2024^19^  Oakley CI 2025^54^  Beyeler M 2023^55^, Fukunaga D 2024^56^ |
| Serological biomarkers | - Cancer-associated ischemic stroke is associated with   - Elevated D-dimer levels   - Elevated D-dimer levels correlate with     - pattern of multiple vascular territories     - embolic signals     - adenocarcinoma histology and systemic metastases   - Elevated fibrinogen and fibrin degradation products   - C-reactive protein levels   - Lower hemoglobin and hematocrit values   - Increased levels of plasma neutrophil count   - CA125 and α-fetoprotein tumor markers | Kim SG 2010^47^, Schwarzbach CJ 2012^9^, Schwarzbach CJ 2015^25^, Gon Y 2016^16^, Bao L 2020^57^, Guo L 2020^48^, Tanimura J 2022^22^, Xie L 2025^58^  Nezu T 2022^14^, Guo YJ 2014^51^, Wang J 2018^52^  Seok JM 2010^59^  Lee MJ 2017^60^  Kono T 2012^61^, Guo YJ 2014^51^, Wang J 2018^52^, Selvik HA 2018^1^  Acir I 2024^8^  Bravo-Anguiano Y 2023^17^, Kneihsl M 2016^12^, Cocho D 2015^3^, Xie L 2025^58^  Nam K-W 2018^62^, Kawano T 2021^63^, Cen G 2023^28^  Maezono-Kandori K 2021^64^, Nezu T 2022^14^, Cen G 2023^28^ |
| Treatment and prevention | - Antithrombotic therapy after ATE is associated with improved survival among patients with cancer - In cancer-associated stroke patients, treatment with enoxaparin may be more effective than treatment with warfarin for lowering the D-dimer levels - Major bleeding, thromboembolic events, and survival were not significantly different with enoxaparin or aspirin in patients with active cancer and stroke - DOAC showed similar clinical outcomes and safety compared with LMWH in the treatment of cryptogenic ischemic stroke in active cancer patients - No difference in major or minor bleeds or thromboembolic events comparing DOAC to enoxaparin or DOAC to warfarin - Oral edoxaban performed similarly to subcutaneous enoxaparin in cancer-associated hypercoagulability - Major ischemic and hemorrhagic events did not differ with apixaban compared with aspirin in patients with history of cancer and cryptogenic stroke - No differences in mortality or recurrences were found in patients with stroke and active cancer treated with anticoagulant therapy versus antiplatelet therapy | Lee MJ 2017^60^, Gon Y 2024^2^  Jang H 2015^65^  Navi BB 2018^66^  Nam K-W 2017^67^  Xiang E 2018^68^  Chung JW 2024^69^  Navi BB 2024^70^  Kielkopf MC 2025^71^ |
| Prognosis | - Hypercoagulability is associated with higher in-hospital mortality and reduced survival in patients with active cancer and ischemic stroke - Half of patients with cancer and stroke die within 3 months, and functional outcome is poor for 50% of 3-month survivors despite acute stroke treatment - During the 6-month follow-up, 90% of patients with NBTE died or developed recurrent stroke - D- dimer levels, systemic metastases, and diabetes are independent predictors of poor survival in cancer patients with stroke - High plasma D-dimer levels after anticoagulant therapy are associated with poor short-term outcomes and recurrent stroke in patients with cancer-associated stroke. - Elevated vWF antigen levels are associated with adverse outcomes in patients with cancer-associated stroke - A fibrin-platelet proportion of ≥79.4% in thrombi could predict stroke recurrence or mortality at 90 days after stroke in patients with active cancer receiving EVT - Elevated TF activity is independently associated with 1-year mortality in patients with active cancer and stroke | Lee MJ 2017^60^, Costamagna G 2023^15^, Schwarzbach CJ 2012^9^, Navi BB 2014^11^, Kneihsl M 2016^12^, Gon Y 2023^72^  Cutting S 2017^73^, Taccone FS 2008^23^, Ohno M 2023^74^, Zhao B 2024^19^, Chandu M 2025^26^  Yoo J 2020^29^  Nam K-W 2017^75^, Navi BB 2022^32^, Fujinami J 2023^76^, Shin YW 2016^77^, Nakajima S 2022^13^, Bravo-Anguiano Y 2023^17^, Chandu 2025^26^, Ke X 2025^78^  Nakajima S 2022^13^, Fujinami J 2023^76^, Kim TJ 2025^79^  Kawano T 2024^80^  Fu CH 2025^81^  Gon Y 2026^82^ |

*ATE, arterial thromboembolism; NBT, non-bacterial thrombotic endocarditis; TF, tissue factor; p53, tumor suppressor gene p53; KRAS, Kirsten rat sarcoma viral oncogene homolog; STK11, serine/threonine kinase 11; FVIII, coagulation factor VIII; FIX, coagulation factor IX; NETosis, neutrophil extracellular trap formation; H3Cit, citrullinated histone H3; DNA, deoxyribonucleic acid; EVs, extracellular vesicles; vWF, von Willebrand factor; IGHG1, immunoglobulin heavy constant gamma 1; VTN, vitronectin; MRI, magnetic resonance imaging; CA-125, cancer antigen 125; DOAC, direct oral anticoagulant; LMWH, low-molecular-weight heparin; EVT, endovascular treatment.*

**REFERENCES:**

1.	Selvik HA, Bjerkreim AT, Thomassen L, Waje-Andreassen U, Naess H, Kvistad CE. When to Screen Ischaemic Stroke Patients for Cancer. *Cerebrovasc Dis*. 2018;45(1-2):42-47. doi:10.1159/000484668

2.	Gon Y, Morishima T, Kawano T, et al. Arterial Thromboembolism in Japanese Patients With Cancer: Incidence, Predictors, and Survival Impact. *JACC CardioOncology*. 2024;6(2):283-297. doi:10.1016/j.jaccao.2024.01.006

3.	Cocho D, Gendre J, Boltes A, et al. Predictors of occult cancer in acute ischemic stroke patients. *J stroke Cerebrovasc Dis Off J Natl Stroke Assoc*. 2015;24(6):1324-1328. doi:10.1016/j.jstrokecerebrovasdis.2015.02.006

4.	Oh MS, Yu KH, Lee BC, et al. Temporal patterns of occult cancer incidence following acute ischemic stroke: A comparative study by stroke etiology. *Int J stroke Off J Int Stroke Soc*. 2025;20(8):1031-1041. doi:10.1177/17474930251335615

5.	Zöller B, Ji J, Sundquist J, Sundquist K. Risk of haemorrhagic and ischaemic stroke in patients with cancer: a nationwide follow-up study from Sweden. *Eur J Cancer*. 2012;48(12):1875-1883. doi:10.1016/j.ejca.2012.01.005

6.	Navi BB, Reiner AS, Kamel H, et al. Association between incident cancer and subsequent stroke. *Ann Neurol*. 2015;77(2):291-300. doi:10.1002/ana.24325

7.	Quintas S, Rogado J, Gullón P, et al. Predictors of unknown cancer in patients with ischemic stroke. *J Neurooncol*. 2018;137(3):551-557. doi:10.1007/s11060-017-2741-0

8.	Acır İ, Erdoğan HA, Toka E, Yayla V. The Influence of Cancer Type on Stroke Incidence: A Retrospective Study. *Clin Med Insights Oncol*. 2024;18:11795549241308090. doi:10.1177/11795549241308090

9.	Schwarzbach CJ, Schaefer A, Ebert A, et al. Stroke and cancer: the importance of cancer-associated hypercoagulation as a possible stroke etiology. *Stroke*. 2012;43(11):3029-3034. doi:10.1161/STROKEAHA.112.658625

10.	Navi BB, Singer S, Merkler AE, et al. Cryptogenic subtype predicts reduced survival among cancer patients with ischemic stroke. *Stroke*. 2014;45(8):2292-2297. doi:10.1161/STROKEAHA.114.005784

11.	Navi BB, Singer S, Merkler AE, et al. Recurrent thromboembolic events after ischemic stroke in patients with cancer. *Neurology*. 2014;83(1):26-33. doi:10.1212/WNL.0000000000000539

12.	Kneihsl M, Enzinger C, Wünsch G, et al. Poor short-term outcome in patients with ischaemic stroke and active cancer. *J Neurol*. 2016;263(1):150-156. doi:10.1007/s00415-015-7954-6

13.	Nakajima S, Kawano H, Yamashiro K, et al. Post-Treatment Plasma D-Dimer Levels Are Associated With Short-Term Outcomes in Patients With Cancer-Associated Stroke. *Front Neurol*. 2022;13(April):1-9. doi:10.3389/fneur.2022.868137

14.	Nezu T, Hosomi N, Naito H, et al. Clinical characteristics and tumor markers in ischemic stroke patients with active cancer. *Intern Emerg Med*. 2022;17(3):735-741. doi:10.1007/s11739-021-02862-1

15.	Costamagna G, Hottinger A, Milionis H, et al. Clinical and Demographic Characteristics, Mechanisms, and Outcomes in Patients With Acute Ischemic Stroke and Newly Diagnosed or Known Active Cancer. *Neurology*. 2023;100(24):E2477-E2489. doi:10.1212/WNL.0000000000207341

16.	Gon Y, Okazaki S, Terasaki Y, et al. Characteristics of cryptogenic stroke in cancer patients. *Ann Clin Transl Neurol*. 2016;3(4):280-287. doi:10.1002/acn3.291

17.	Bravo-anguiano Y, Echavarría-iñiguez A, Madrigal-lkhou E, Muñoz-martín A. Ictus asociado a cáncer : estudio de prevalencia y factores predictores entre pacientes con ictus isquémico. 2023;76(6):189-195. doi:10.33588/rn.7606.2022414.

18.	Patrzalek P, Wysokinski WE, Kurmann RD, et al. Cancer-associated non-bacterial thrombotic endocarditis-Clinical series from a single institution. *Am J Hematol*. 2024;99(4):596-605. doi:10.1002/ajh.27239

19.	Zhao B, Jia W, Yuan Y, Li Z. Clinical analysis of twenty ‑ one cases of acute ischemic stroke related to Trousseau syndrome. *Neurol Sci*. 2024;45(4):1537-1547. doi:10.1007/s10072-023-07180-2

20.	Oka K, Iwai N, Ohara T, et al. Ischemic stroke in patients with unresectable pancreatic and gastric cancer. *Int J Clin Oncol*. 2025;30(5):974-983. doi:10.1007/s10147-025-02731-3

21.	Seok JM, Kim SJ, Song P, et al. Clinical presentation and ischemic zone on MRI in cancer patients with acute ischemic stroke. *Eur Neurol*. 2012;68(6):368-376. doi:10.1159/000341147

22.	Tanimura J, Yamamoto T, Hashimoto T. Clinical features and prognostic factors in patients with cancer-associated multiple ischemic stroke: A retrospective observational study. *J stroke Cerebrovasc Dis Off J Natl Stroke Assoc*. 2022;31(12):106813. doi:10.1016/j.jstrokecerebrovasdis.2022.106813

23.	Taccone FS, Jeangette SM, Blecic SA. First-Ever Stroke as Initial Presentation of Systemic Cancer. *J Stroke Cerebrovasc Dis*. 2008;17(4):169-174. doi:10.1016/j.jstrokecerebrovasdis.2008.01.007

24.	Ren R, Yan H, Gui Y, et al. Clinical Features of Trousseau Syndrome With Cerebral Infarction as the Initial Manifestation. *Neurologist*. 2020;25(5):117-121. doi:10.1097/NRL.0000000000000275

25.	Schwarzbach CJ, Fatar M, Eisele P, Ebert AD, Hennerici MG, Szabo K. DWI lesion patterns in cancer-related stroke – specifying the phenotype. *Cerebrovasc Dis Extra*. 2015;5(3):139-145. doi:10.1159/000439549

26.	Chandu M, Agarwal A, Vishnu VY, et al. Patterns and predictors of cancer-associated ischemic stroke. *J Neurosci Rural Pract*. 2025;16. doi:10.25259/JNRP_88_2025

27.	Zhang J, Zhao J. Clinical Characteristics and Analysis of Lung Cancer- Associated Acute Ischemic Stroke. *J stroke Cerebrovasc Dis Off J Natl Stroke Assoc*. 2020;29(10):105164. doi:10.1016/j.jstrokecerebrovasdis.2020.105164

28.	Cen G, Song Y, Chen S, et al. The investigation on the hypercoagulability of hepatocellular carcinoma-related cerebral infarction with thromboelastography. *Brain Behav*. 2023;13(4):e2961. doi:10.1002/brb3.2961

29.	Yoo J, Choi JK, Kim YD, et al. Outcome of Stroke Patients with Cancer and Nonbacterial Thrombotic Endocarditis. *J Stroke*. 2020;22(2):245-253. doi:10.5853/jos.2020.00619

30.	Grazioli S, Paciaroni M, Agnelli G, et al. Cancer-associated ischemic stroke: A retrospective multicentre cohort study. *Thromb Res*. 2018;165:33-37. doi:10.1016/j.thromres.2018.03.011

31.	Karlińska AG, Gromadzka G, Karliński MA, Członkowska A. The activity of malignancy may determine stroke pattern in cancer patients. *J stroke Cerebrovasc Dis Off J Natl Stroke Assoc*. 2015;24(4):778-783. doi:10.1016/j.jstrokecerebrovasdis.2014.11.003

32.	Navi BB, Zhang C, Sherman CP, et al. Ischemic stroke with cancer: Hematologic and embolic biomarkers and clinical outcomes. *J Thromb Haemost*. 2022;20(9):2046-2057. doi:10.1111/jth.15779

33.	Cen G, Wang J, Wang X, et al. Pathogenesis and Biomarkers of Cancer-Related Ischemic Stroke. *J Inflamm Res*. 2024;17:8589-8597. doi:10.2147/JIR.S493406

34.	Park H, Kim J, Ha J, et al. Histological features of intracranial thrombi in stroke patients with cancer. *Ann Neurol*. 2019;86(1):143-149. doi:10.1002/ana.25495

35.	Kataoka Y, Sonoda K, Takahashi JC, et al. Histopathological analysis of retrieved thrombi from patients with acute ischemic stroke with malignant tumors. *J Neurointerv Surg*. 2022;14(5). doi:10.1136/neurintsurg-2020-017195

36.	Fu CH, Chen CH, Lin YH, et al. Fibrin and Platelet-Rich Composition in Retrieved Thrombi Hallmarks Stroke With Active Cancer. *Stroke*. 2020;51(12):3723-3727. doi:10.1161/STROKEAHA.120.032069

37.	Otani T, Terada-Ikeda C, Koge J, et al. Vegetations and cerebral emboli of non-bacterial thrombotic endocarditis are overwhelmingly platelet-dominant: A possible histological clue for the diagnosis from an autopsy study. *Cardiovasc Pathol*. 2025;78:107746. doi:https://doi.org/10.1016/j.carpath.2025.107746

38.	Feldman S, Gupta D, Navi BB, et al. Tumor Genomic Profile Is Associated With Arterial Thromboembolism Risk in Patients With Solid Cancer. *JACC CardioOncology*. 2023;5(2):246-255. doi:10.1016/j.jaccao.2023.01.009

39.	Chou SC, Chang SL, Yu CY, et al. Tissue factor, factor VIII and IX in microvesicle-induced thrombosis and tumor growth of pancreatic cancer. *Thromb J*. 2025;23(1):32. doi:10.1186/s12959-025-00715-x

40.	Thålin C, Demers M, Blomgren B, et al. NETosis promotes cancer-associated arterial microthrombosis presenting as ischemic stroke with troponin elevation. *Thromb Res*. 2016;139:56-64. doi:10.1016/j.thromres.2016.01.009

41.	Bang OY, Chung JW, Cho YH, et al. Circulating DNAs, a Marker of Neutrophil Extracellular Traposis and Cancer-Related Stroke: The OASIS-Cancer Study. *Stroke*. 2019;50(10):2944-2947. doi:10.1161/STROKEAHA.119.026373

42.	Hyun JW, Payumo RMP, Chung J, et al. Neutrophil extracellular traposis in cancer patients with acute ischemic stroke. *PLoS One*. 2025;20(6):e0326654. doi:10.1371/journal.pone.0326654

43.	Bang OY, Chung JW, Lee MJ, et al. Cancer cell-derived extracellular vesicles are associated with coagulopathy causing ischemic stroke via tissue factor-independent way: The OASIS-CANCER study. *PLoS One*. 2016;11(7):1-15. doi:10.1371/journal.pone.0159170

44.	Chung JW, Cho YH, Ahn MJ, et al. Association of cancer cell type and extracellular vesicles with coagulopathy in patients with lung cancer and stroke. *Stroke*. 2018;49(5):1282-1285. doi:10.1161/STROKEAHA.118.020995

45.	Woock M, Rossi R, Jabrah D, et al. Clot signature in patients with large vessel occlusion stroke and concomitant active cancer. *Eur J Neurol*. 2025;32(1):e70037. doi:10.1111/ene.70037

46.	Kim TJ, Jung JW, Kim YJ, Yoon BW, Han D, Ko SB. Proteomic Analyses of Clots Identify Stroke Etiologies in Patients Undergoing Endovascular Therapy. *CNS Neurosci Ther*. 2025;31(3):e70340. doi:10.1111/cns.70340

47.	Kim SG, Hong JM, Kim HY, et al. Ischemic stroke in cancer patients with and without conventional mechanisms: a multicenter study in Korea. *Stroke*. 2010;41(4):798-801. doi:10.1161/STROKEAHA.109.571356

48.	Guo L, Wang L, Liu W. Ability of the number of territories involved on DWI-MRI to predict occult systemic malignancy in cryptogenic stroke patients. *J Stroke Cerebrovasc Dis*. 2020;29(7):104823. doi:10.1016/j.jstrokecerebrovasdis.2020.104823

49.	Finelli PF, Nouh A. Three-Territory DWI acute infarcts: Diagnostic value in cancer-Associated hypercoagulation stroke (trousseau syndrome). *Am J Neuroradiol*. 2016;37(11):2033-2036. doi:10.3174/ajnr.A4846

50.	Finelli PF. MR Three-Territory Sign in Cancer-Associated Hypercoagulation Stroke (Trousseau Syndrome): An Overlooked Diagnostic Feature: MR Three-Territory Sign. *Neurologist*. 2022;27(2):37-40. doi:10.1097/NRL.0000000000000356

51.	Guo YJ, Chang MH, Chen PL, Lee YS, Chang YC, Liao YC. Predictive value of plasma (D)-dimer levels for cancer-related stroke: a 3-year retrospective study. *J stroke Cerebrovasc Dis Off J Natl Stroke Assoc*. 2014;23(4):e249-54. doi:10.1016/j.jstrokecerebrovasdis.2013.10.022

52.	Wang J ye, Zhang G jia, Zhuo S xia, et al. D-dimer >2.785 μg/ml and multiple infarcts ≥3 vascular territories are two characteristics of identifying cancer-associated ischemic stroke patients. *Neurol Res*. 2018;40(11):948-954. doi:10.1080/01616412.2018.1504179

53.	Nouh AM, Staff I, Finelli PF. Three Territory Sign: An MRI marker of malignancy-related ischemic stroke (Trousseau syndrome). *Neurol Clin Pract*. 2019;9(2):124-128. doi:10.1212/CPJ.0000000000000603

54.	Oakley CI, Wysokinska EM, Kaminska A, et al. Topography of ischemic strokes in cancer-associated non-bacterial thrombotic endocarditis: A single-institution descriptive case series. *J stroke Cerebrovasc Dis Off J Natl Stroke Assoc*. 2025;34(2):108171. doi:10.1016/j.jstrokecerebrovasdis.2024.108171

55.	Beyeler M, Grunder L, Göcmen J, et al. Absence of susceptibility vessel sign and hyperdense vessel sign in patients with cancer-related stroke. *Front Neurol*. 2023;14(March):1-8. doi:10.3389/fneur.2023.1148152

56.	Fukunaga D, Fujinami J, Kishitani T, Tokuda N, Numa S, Nagakane Y. Absence of the Susceptibility Vessel Sign with Cancer-Associated Hypercoagulability-Related Stroke. *AJNR Am J Neuroradiol*. 2024;45(10):1427-1431. doi:10.3174/ajnr.A8363

57.	Bao L, Zhang S, Gong X, Cui G. Trousseau Syndrome Related Cerebral Infarction: Clinical Manifestations, Laboratory Findings and Radiological Features. *J stroke Cerebrovasc Dis Off J Natl Stroke Assoc*. 2020;29(9):104891. doi:10.1016/j.jstrokecerebrovasdis.2020.104891

58.	Xie L, Hao K, Shen J, Huang C. Association of red blood cells, D-dimer, and 13-(S)-HODE with cancer-related ischemic stroke: a case-control study. *Am J Cancer Res*. 2025;15(9):4054-4066. doi:10.62347/RHLH9776

59.	Seok JM, Kim SG, Kim JW, et al. Coagulopathy and embolic signal in cancer patients with ischemic stroke. *Ann Neurol*. 2010;68(2):213-219. doi:10.1002/ana.22050

60.	Lee MJ, Chung JW, Ahn MJ, et al. Hypercoagulability and Mortality of Patients with Stroke and Active Cancer: The OASIS-CANCER Study. *J stroke*. 2017;19(1):77-87. doi:10.5853/jos.2016.00570

61.	Kono T, Ohtsuki T, Hosomi N, et al. Cancer-associated ischemic stroke is associated with elevated D-dimer and fibrin degradation product levels in acute ischemic stroke with advanced cancer. *Geriatr Gerontol Int*. 2012;12(3):468-474. doi:10.1111/j.1447-0594.2011.00796.x

62.	Nam KW, Kim TJ, Kim CK, et al. Temporal changes in the neutrophil to lymphocyte ratio and the neurological progression in cryptogenic stroke with active cancer. *PLoS One*. 2018;13(3):e0194286. doi:10.1371/journal.pone.0194286

63.	Kawano T, Sasaki T, Gon Y, et al. High neutrophil/lymphocyte ratio at cancer diagnosis predicts incidence of stroke in cancer patients. *Brain Commun*. 2021;3(2):fcab071. doi:10.1093/braincomms/fcab071

64.	Maezono-Kandori K, Ohara T, Fujinami J, Makita N, Tanaka E, Mizuno T. Elevated CA125 is Related to Stroke Due to Cancer- Associated Hypercoagulation. *J Stroke Cerebrovasc Dis*. 2021;30(12):106126. doi:10.1016/j.jstrokecerebrovasdis.2021.106126

65.	Jang H, Lee JJ, Lee MJ, et al. Comparison of Enoxaparin and Warfarin for Secondary Prevention of Cancer-Associated Stroke. *J Oncol*. 2015;2015(July 2006). doi:10.1155/2015/502089

66.	Navi BB, Marshall RS, Bobrow D, et al. Enoxaparin vs Aspirin in Patients With Cancer and Ischemic Stroke: The TEACH Pilot Randomized Clinical Trial. *JAMA Neurol*. 2018;75(3):379-381. doi:10.1001/jamaneurol.2017.4211

67.	Nam KW, Kim CK, Kim TJ, et al. Treatment of Cryptogenic Stroke with Active Cancer with a New Oral Anticoagulant. *J stroke Cerebrovasc Dis Off J Natl Stroke Assoc*. 2017;26(12):2976-2980. doi:10.1016/j.jstrokecerebrovasdis.2017.07.029

68.	Xiang E, Ahuja T, Raco V, Cirrone F, Green D, Papadopoulos J. Anticoagulation prescribing patterns in patients with cancer. *J Thromb Thrombolysis*. 2018;45(1):89-98. doi:10.1007/s11239-017-1558-1

69.	Chung JW, Hwang J, Kim HJ, et al. Edoxaban for the treatment of hypercoagulability and cerebral thromboembolism associated with cancer: A randomized clinical trial of biomarker targets. *Int J Stroke*. 2024;19(6):645-653. doi:10.1177/17474930241239266

70.	Navi BB, Zhang C, Miller B, et al. Apixaban vs Aspirin in Patients With Cancer and Cryptogenic Stroke: A Post Hoc Analysis of the ARCADIA Randomized Clinical Trial. *JAMA Neurol*. 2024;81(9):958-965. doi:10.1001/jamaneurol.2024.2404

71.	Kielkopf MC, Göcmen J, Venzin SB, et al. Anticoagulant versus antiplatelet treatment for secondary stroke prevention in patients with active cancer. *Front Neurol*. 2025;Volume 16-2025. doi:10.3389/fneur.2025.1530775

72.	Gon Y, Sakaguchi M, Yamagami H, et al. Predictors of Survival in Patients With Ischemic Stroke and Active Cancer: A Prospective, Multicenter, Observational Study. *J Am Heart Assoc*. 2023;12(15):e029618. doi:10.1161/JAHA.123.029618

73.	Cutting S, Wettengel M, Conners JJ, Ouyang B, Busl K. Three-Month Outcomes Are Poor in Stroke Patients with Cancer Despite Acute Stroke Treatment. *J Stroke Cerebrovasc Dis*. 2017;26(4):809-815. doi:10.1016/j.jstrokecerebrovasdis.2016.10.021

74.	Ohno M, Miyakita Y, Takahashi M, Yanagisawa S, Ozeki Y, Yonezawa H, Omura T, Satomi N, Mukai T, Kawaguchi Y NY. Assessment of clinical and functional outcome in patients with cancer and acute ischemic stroke. *Res Sq*. Published online 2023:1-15.

75.	Nam KW, Kim CK, Kim TJ, et al. D-dimer as a predictor of early neurologic deterioration in cryptogenic stroke with active cancer. *Eur J Neurol*. 2017;24(1):205-211. doi:10.1111/ene.13184

76.	Fujinami J, Nagakane Y, Fujikawa K, et al. D-dimer trends predict recurrent stroke in patients with cancer-related hypercoagulability. *Cerebrovasc Dis Extra*. Published online 2023. doi:10.1159/000535644

77.	Shin YW, Lee ST, Jung KH, et al. Predictors of survival for patients with cancer after cryptogenic stroke. *J Neurooncol*. 2016;128(2):277-284. doi:10.1007/s11060-016-2106-0

78.	Ke X, Wu X, Feng G, et al. Analysis of Prognostic Factors for Survival in Patients with Trousseau Syndrome Initially Presenting with Acute Ischemic Stroke. *Cerebrovasc Dis Extra*. 2025;15(1):202-209. doi:10.1159/000548109

79.	Kim TJ, Lee JS, Oh MS, et al. Long-Term Outcomes and Predictors in Cancer-Related Stroke Using the Linked Data Set. *J Am Heart Assoc*. 2025;14(16):e040883. doi:10.1161/JAHA.124.040883

80.	Kawano T, Gon Y, Sakaguchi M, et al. Von Willebrand Factor Antigen Levels Predict Poor Outcomes in Patients With Stroke and Cancer: Findings From the Multicenter, Prospective, Observational SCAN Study. *J Am Heart Assoc*. 2024;13(3):e032284. doi:10.1161/JAHA.123.032284

81.	Fu CH, Chen CH, Lin YH, et al. High fibrin and platelet clot predicts stroke recurrence or mortality after thrombectomy in patients with active cancer. *J Neurointerv Surg*. 2025;17(11):1189-1194. doi:10.1136/jnis-2024-022033

82.	Gon Y, Kawano T, Kitano T, et al. Tissue factor activity and 1-year mortality in patients with active cancer and acute ischemic stroke: findings from the SCAN study. *Res Pract Thromb Haemost*. 2026;10(1):103347. doi:10.1016/j.rpth.2026.103347
